# Silapropofol: Carbon–Silicon Isosterism in a Key Anesthetic Scaffold

**DOI:** 10.1021/acsomega.5c11217

**Published:** 2026-01-07

**Authors:** Sarah Koschabek, Florian Kleemiss, Noel Angel Espinosa-Jalapa, Jonathan O. Bauer

**Affiliations:** † Faculty of Chemistry and Pharmacy, Institute of Inorganic Chemistry, 199842 University of Regensburg, Universitätsstraße 31, D-93053 Regensburg, Germany; ‡ Department of Chemistry, Institute of Inorganic Chemistry, 9165 RWTH Aachen University, Landoltweg 1a, D-52074 Aachen, Germany

## Abstract

Propofol (2,6-di-*iso*-propylphenol) (**1**) is one of the most widely used intravenous anesthetics, yet its high lipophilicity, formulation challenges, and incompletely understood binding mode motivate the exploration of structural analogues. Here, we report the synthesis and comprehensive characterization of the first silicon analogues of propofol, monosilapropofol (**2**) and disilapropofol (**3**), in which one or both *iso*-propyl groups are replaced by dimethylsilyl substituents. Key steps involve optimized [1,3]-retro-Brook rearrangements, with *tert*-butyllithium-mediated Li/Br exchange enabling efficient access to both targets. Crystalline potassium phenolate **2-K** provided the first X-ray diffraction analysis of a silapropofol derivative, and complementary quantum chemical analysis based on orbital, topological, and localizability descriptors revealed pronounced polarization effects and bond umpolung in this pharmacologically relevant scaffold arising from carbon–silicon isosterism. Stability studies under physiological conditions uncovered a strong divergence between the two analogues: while **2** undergoes gradual hydrolysis to 2-*iso*-propylphenol and dimethylsilanol, **3** proved remarkably robust in neutral saline solution. These findings demonstrate that silicon substitution offers a powerful strategy to modulate both electronic properties and aqueous stability in propofol derivatives, highlighting carbon–silicon isosterism as a valuable concept for anesthetic drug design.

## Graphical abstract

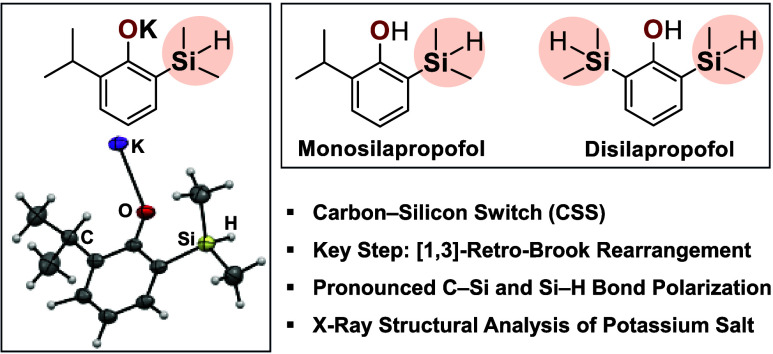

## Introduction

Propofol (2,6-di-*iso*-propylphenol) (**1**, Figure 1) has been one of the most widely used intravenous anesthetics since its clinical introduction in the 1980s, and it is included in the World Health Organization’s List of Essential Medicines.^1,2^ It is routinely administered for the induction and maintenance of general anesthesia as well as for sedation in intensive care units, and it has gained particular importance during the COVID-19 pandemic in the context of long-term mechanical ventilation.^3,4^ Propofol is valued for its rapid onset of action, smooth recovery profile, and relatively low incidence of postoperative nausea and vomiting. However, it lacks intrinsic analgesic activity and is associated with disadvantages such as severe injection pain and formulation challenges arising from its high lipophilicity and poor aqueous solubility.^5–7^

**Figure 1:**
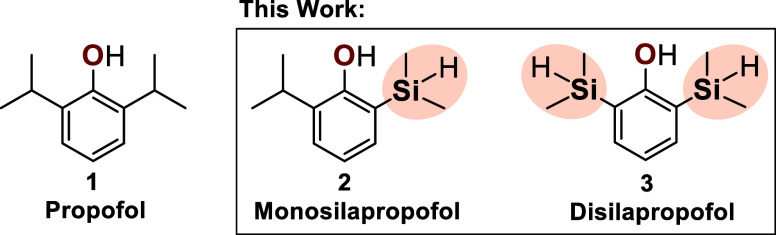
Concept of the carbon–silicon switch in propofol (**1**): replacement of one or both *iso*-propyl groups by dimethylsilyl groups affords the bioisosteric analogues monosilapropofol (**2**) and disilapropofol (**3**) synthesized in this study.

At the molecular level, propofol exerts its pharmacological effects primarily by allosteric modulation of γ-aminobutyric acid type A (GABA_A_) receptors, the most important inhibitory ion channels in the central nervous system.^8,9^ Structural studies have revealed that propofol binds within cavities of the transmembrane domain, where the *iso*-propyl substituents engage in van der Waals interactions and the phenolic hydroxy group forms hydrogen bonds to the receptor backbone.^10–13^ Despite significant progress, the precise mechanisms of receptor modulation remain incompletely understood, underscoring the importance of systematic structure–activity investigations and the design of structurally modified analogues.

In this context, carbon–silicon isosterism has emerged as a powerful concept to probe and modulate molecular properties. The systematic replacement of a carbon atom by silicon—referred to as the carbon–silicon switch (CSS)—preserves the overall molecular framework due to the valence isoelectronic relationship of the two elements, while subtly increasing molecular volume as a result of the larger atomic radius of silicon.^14,15^ More profound, however, are the electronic consequences: the lower electronegativity of silicon compared to carbon leads to altered bond polarities, including an inversion (umpolung) of the bond dipole in Si–H relative to C–H bonds.^16^ Such differences in polarity and polarizability can significantly influence solvation behavior and molecular recognition in protein binding pockets, ultimately affecting pharmacological potency, selectivity, and pharmacokinetic properties.^14^

Although applications of sila analogues in medicinal chemistry are still comparatively limited, they have proven to be valuable tools for tuning biological properties and gaining insights into ligand–receptor interactions and metabolic stability. The potential of CSS has been demonstrated in several cases, including sila-ibuprofen,^17^ sila-haloperidol,^18^ sila-venlafaxine,^19^ disila-bexarotene,^20^ sila-loperamide,^21^ and sila-1,4-dihydropyridine.^22^ These examples illustrate how strategic incorporation of silicon into established drug scaffolds can enhance bioactivity or reveal novel pharmacological profiles.

Herein, we report the first synthesis of sila analogues of propofol (Figure 1). By replacing one or both *iso*-propyl substituents with dimethylsilyl groups, we have prepared monosilapropofol (**2**) and disilapropofol (**3**). Compound **2** was characterized by single-crystal X-ray diffraction analysis in the form of its potassium salt and by quantum chemical methods (QTAIM, NPA, NRT, ESP), enabling a detailed analysis of the structural and electronic consequences of C/Si substitution. Furthermore, the stability of the sila analogues **2** and **3** in aqueous media under physiological conditions was investigated, providing first insights into their potential as novel anesthetic agents.

## Results and Discussion

### Synthesis of Monosilapropofol (**2**)

The synthesis of 2-(dimethylsilyl)-6-*iso*-propylphenol (**2**, monosilapropofol) was accomplished in a three-step sequence (Scheme 1).

**Scheme 1:**
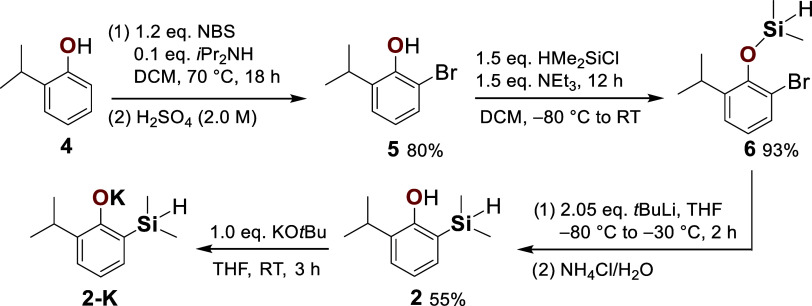
General Synthetic Route to Monosilapropofol (2). Bromination of 2-*iso*-Propylphenol (4) Provides 2-Bromo-6-*iso*-Propylphenol (5). Subsequent Hydroxy Silylation Furnishes the Corresponding Silyl Ether (6), Which Undergoes an Intramolecular [1,3]-Retro-Brook Rearrangement to Deliver Monosilapropofol (2). Deprotonation with Potassium *tert*-Butoxide Affords the Crystalline Potassium Phenolate (2-K)

The initial *ortho*-bromination of 2-*iso*-propylphenol (**4**) proceeded under Soxhlet conditions with *N*-bromosuccinimide (NBS) as the bromine source, following a modified literature protocol.^23^ Control of the NBS stoichiometry proved to be essential for reproducibility: with one equivalent of NBS a conversion of 74% was achieved, whereas a slight excess (1.2 equiv) increased the conversion to 88%. After purification, the isolated material (80%) consisted of 94% 2-bromo-6-*iso*-propylphenol (**5**) and 6% unreacted starting material **4**.

In the second step, silylation of the hydroxy group was performed using chlorodimethylsilane (1.5 equiv) and triethylamine (1.5 equiv) in dichloromethane (DCM). The reaction afforded the expected silyl ether **6** in 94% purity alongside a minor byproduct (6%) of the corresponding silyl ether, which originated from residual **4** in the preceding step. Pure compound **6** was isolated in 93% yield after Kugelrohr distillation.

The key transformation was a [1,3]-retro-Brook rearrangement of compound **6**,^24–28^ enabling migration of the dimethylsilyl moiety from oxygen to the *ortho*-carbon atom (Scheme 1). Initial experiments with one equivalent of *n*-butyllithium in tetrahydrofuran (THF) at −80 °C to −30 °C gave incomplete conversion, yielding 56% of **2** together with 44% of **5**, the latter arising from hydrolytic cleavage of unconverted starting material during workup (for details, see the Supporting Information, SI, Table S1). Direct warming from −80 °C to room temperature and prolonging the reaction time to 24 h further reduced the yield of **2** to 44%. Changing the solvent to diethyl ether applying temperatures from −80 °C to −30 °C gave no conversion. Employing a slight excess of *n*-butyllithium (1.1 equiv) in THF improved the conversion to give 60% of **2**. Significantly better results were obtained with *tert*-butyllithium as a base. When compound **6** was treated with 2.05 equiv of *tert*-butyllithium in THF at −80 °C, followed by gradual warming to −30 °C, the crude mixture contained 92% monosilapropofol (**2**) and only 8% of **5**. This result highlights the higher efficiency of Li/Br exchange under the stronger basicity of *tert*-butyllithium, favoring productive rearrangement.

Attempts to separate **2** from minor amounts of **5** by column chromatography were unsuccessful due to cleavage of the sensitive dimethylsilyl group, even when neutralized silica was employed. While Kugelrohr distillation did not afford significant additional purification, subsequent low-temperature precipitation (−80 °C) from *n*-hexane provided the target compound **2** as an amorphous solid in excellent purity, with an isolated yield of 55 % (Scheme 1). The reaction scale also influenced conversion efficiency: batches above 10 mmol exhibited reduced yields, presumably due to incomplete cooling and diffusion limitations, underscoring the importance of precise low-temperature control.

As monosilapropofol **2** is a liquid above −80 °C, it was unsuitable for single-crystal X-ray diffraction analysis. Although cocrystallization with isonicotinamide is known for propofol,^29^ the same approach with **2** was unsuccessful, likely due to the reduced Lewis basicity of its phenolic hydroxy group caused by the carbon–silicon switch. To overcome this limitation and further probe its reactivity, alkali metal phenolates were prepared. Reaction with sodium *tert*-butoxide yielded only amorphous material, whereas treatment with potassium *tert*-butoxide furnished the crystalline potassium phenolate **2-K**, suitable for single-crystal X-ray diffraction analysis (Figure 2). Structural analysis based on the diffraction data revealed significant elongation of the C–Si bonds by ∼0.33 Å relative to the corresponding C–C bonds of the *iso*-propyl substituent, while bond angles remained essentially unchanged.

**Figure 2:**
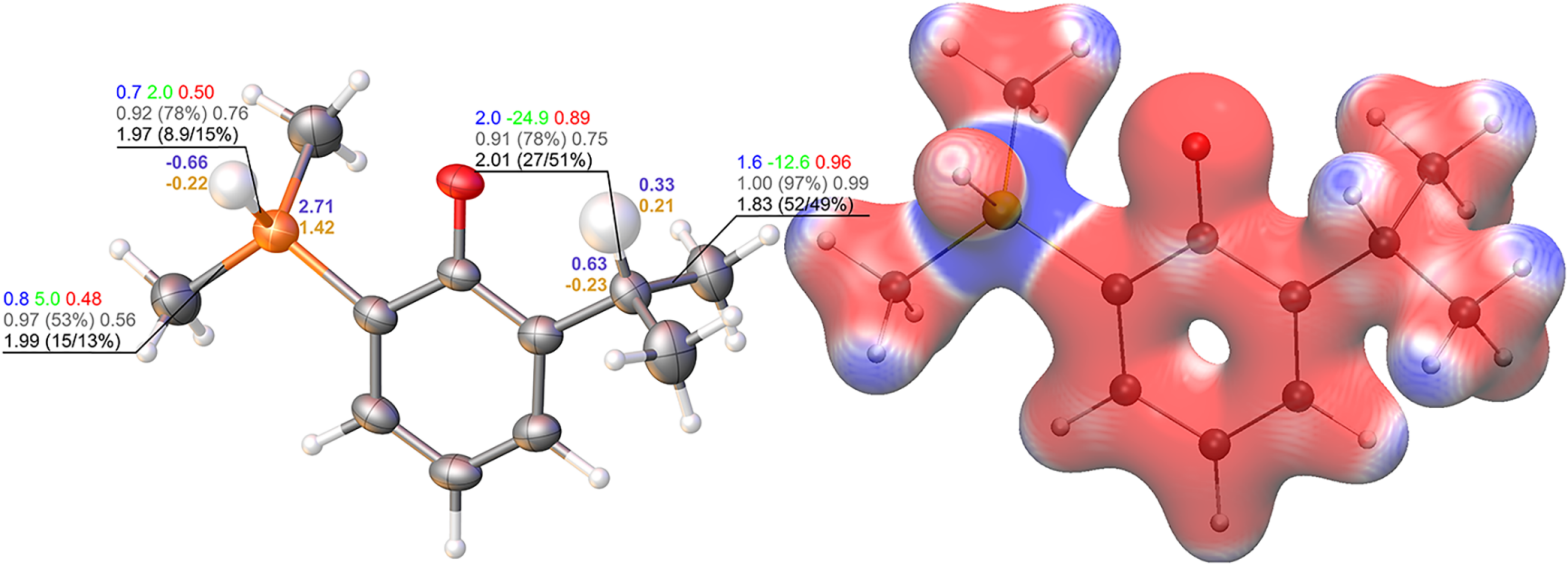
Left: Molecular structure of the potassium salt of the major disorder component of monosilapropofol (**2-K**) in the crystal (only anion shown). Anisotropic displacement parameters (ADPs) of non-hydrogen atoms are shown at the 80% probability level; hydrogen atoms are depicted at an arbitrary scale, with enlarged ADPs highlighting the discussed charge distribution. Bond descriptors are labeled according to the following color code: QTAIM charges (*e*, violet), NPA charges (*e*, yellow), electron density (ρ in eÅ^–3^, blue) and Laplacian of the density (Δρ in eÅ^–5^, green) at the bond critical point (BCP), delocalization index (red), NBO bond order with percentage weight of the covalent portion (in parentheses) and with NLMO/NPA bond order (gray), and population of the diatomic ELI-D basin with contribution from the higher-Z element to the bond given by volume/electron density (black). Right: Electrostatic potential (ESP) mapped onto the 0.001 au electron density isosurface, color-coded from −0.01 au (red) to +0.01 au (blue).

### Bonding Analysis and Electrostatic Properties of 2-K

The carbon–silicon switch (CSS) offers a unique strategy to probe bioisosterism by altering electronic distribution while maintaining steric similarity.^30,31^ Detailed quantum-chemical analyses of the molecular structure of **2-K** (ORCA 5.0, PBE/def2-TZVPPD)^32–37^ provided insight into the electronic consequences of C/Si substitution (Figure 2).^38,39^ Natural population analysis (NPA)^40^ and quantum theory of atoms in molecules (QTAIM)^41^ revealed the expected umpolung upon carbon–silicon switch: the hydrogen atom attached to silicon carried a net negative charge (−0.22 e NPA; −0.66 e QTAIM), in sharp contrast to the positively polarized hydrogen atom of the tertiary carbon atom of the *iso*-propyl group (+0.21 e NPA; + 0.33 e QTAIM). Similarly, the silicon atom exhibited strong positive polarization (1.42 e/2.71 e based on NPA/QTAIM), while the corresponding tertiary carbon atom displayed a weakly negative to slightly positive charge (−0.23 e/0.63 *e* based on NPA/QTAIM).

In addition to the pronounced charge redistribution, the bonding analysis reveals a fundamental difference between C–H and Si–H bonds. QTAIM descriptors (electron density and Laplacian at the bond critical point, and the delocalization index) indicate that the C–H bond in the *iso*-propyl group retains a highly covalent character, whereas the corresponding Si–H bond in the dimethylsilyl group is markedly more polarized, characterized by a positive Laplacian and a delocalization index below 0.5. This trend is corroborated by the Raub–Jansen index (RJI)^42^ of the heavier element, which decreases from 51% for C–H to only 15% for Si–H, highlighting the electron density shift toward the hydrogen atom in the silyl function. A similar dichotomy is observed for the C–C versus Si–C bonds: in the *iso*-propyl fragment, high electron density, negative Laplacian, a delocalization index close to unity, and 97% covalency based on natural resonance theory (NRT)^43^ analyses (49% according to RJI) collectively underscore an unpolarized covalent bonding situation. By contrast, the Si–C bond is substantially more polarized, displaying lower density, a positive Laplacian, a delocalization index of ∼0.5, and only 53% covalency according to NRT, which is further reflected in a significantly reduced RJI (<15%) (Figure 2).

The pronounced umpolung effect introduced by the carbon–silicon switch is also mirrored in the electrostatic potential (ESP) distribution, showing a positive region around the hydrogen atom of the tertiary carbon atom but a negative region around the silicon-bound hydrogen atom. Conversely, the ESP around the tertiary carbon atom is shifted to negative values, while the silicon atom itself adopts a positive potential. This inversion of local electrostatics underscores the unique capacity of carbon–silicon isosterism to tune bond polarity and electronic surface properties without altering the overall steric framework. Such changes are expected to influence interactions with receptor sites substantially.

### Stability of Monosilapropofol (**2**) in Aqueous Solution

The hydrolytic stability of **2** was investigated under simulated physiological conditions (for details, see the SI, Section 2.5). In 0.9% NaCl/D_2_O at room temperature, ^1^H NMR spectra showed a gradual decomposition from 98% purity initially to 78% after 2 weeks and near-complete hydrolysis after 6 weeks. Under mildly basic conditions (10 mM NaHCO_3_/D_2_O, pH ≈ 8), decomposition was significantly faster: After 4 days, only 60% of **2** remained, accompanied by progressively broadened NMR signals, indicative of ongoing decomposition. The NMR spectra do not allow for an unambiguous assignment of the decomposition products. However, the still observable Si–H signal indicates that dimethylsilanol is likely formed as one of the hydrolysis products, together with 2-*iso*-propylphenol and other unidentified side products.

### Synthesis of Disilapropofol (**3**)

Encouraged by the successful synthesis of **2**, we pursued the preparation of 2,6-bis­(dimethylsilyl)­phenol (**3**, disilapropofol) (Scheme 2). Irrespective of the specific pharmacological relevance of disilapropofol, 2,6-bis­(trimethylsilyl)­phenyl triflates have recently attracted considerable attention as precursors to silylbenzynes, which display unique and remarkable reactivity.^44^

**Scheme 2:**
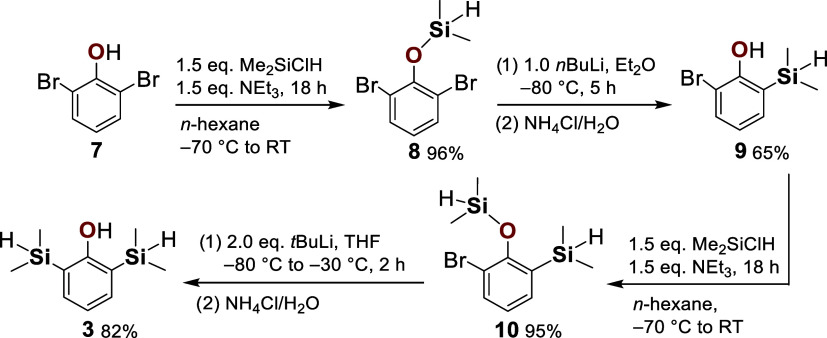
General Synthetic Route to Disilapropofol (3). Successive Hydroxy Silylation and [1,3]-Retro-Brook Rearrangement Enable Exchange of Both Bromine Substituents in 2,6-Dibromophenol (7), Ultimately Affording Disilapropofol (3)

The sequence commenced with the silylation of 2,6-dibromophenol (**7**), furnishing the corresponding dimethylsilyl ether **8** in 96% isolated yield. Subsequent [1,3]-retro-Brook rearrangement of **8** provided 2-bromo-6-(dimethylsilyl)­phenol (**9**), with the conversion strongly dependent on base and solvent (for details, see the SI, Table S2). Treatment with *tert*-butyllithium promoted double Li/Br exchange, leading not only to the formation of compound **9** but also to 2-(dimethylsilyl)­phenol as a byproduct, consistent with overlithiation. Analogous to the behavior observed with *tert*-butyllithium, diethyl ether afforded higher conversion to **9** (66%) than THF (25%) at low temperature (−80 °C to −30 °C), which can be rationalized by the weaker stabilization of multiply charged intermediates in diethyl ether, thereby suppressing excessive Li/Br exchange and byproduct formation. By contrast, reactions with *n*-butyllithium resulted in only a single Li/Br exchange, giving **9** alongside unreacted **7** after aqueous workup. The best conversion to **9** (82%) was achieved in diethyl ether at low temperature (−80 °C), whereas THF under prolonged room temperature conditions led to considerably lower conversion (36%). Crude mixtures were subjected to Kugelrohr distillation; however, complete separation was not feasible due to the nearly identical molecular weights and boiling points of the components. Under the optimized conditions, a mixture of **9** (86%) and **7** (14%) was obtained as a colorless liquid after distillation. Subsequent recrystallization from *n*-hexane at −80 °C afforded colorless needles of **7** ^45^ and, in this way, pure compound **9** in 65% isolated yield (Scheme 2).

Silylation of **9** under standard conditions afforded the pure silyl ether **10** in 95% isolated yield. The final step toward disilapropofol (**3**) involved a [1,3]-retro-Brook rearrangement of **10**. Under the conditions previously optimized for the monosilyl analogue **9** (*n*-butyllithium in diethyl ether, −80 °C), conversion to **3** proved minimal (4%). In contrast, treatment of **10** with *tert*-butyllithium (2.0 equiv) in THF at −80 °C, followed by warming to −30 °C, furnished a mixture of **3** (97%), **9** (1%), and 2-(dimethylsilyl)­phenol (2%), from which pure compound **3** was isolated in 82% yield after Kugelrohr distillation (Scheme 2).

### Stability of Disilapropofol (**3**) in Aqueous Solution

The aqueous stability of **3** was examined in direct analogy to **2** (for details, see the SI, Section 2.10). Remarkably, **3** displayed superior stability under neutral conditions: no significant decomposition was observed in 0.9% NaCl/D_2_O at room temperature over 6 weeks. Under basic conditions (10 mM NaHCO_3_/D_2_O), however, decomposition occurred more rapidly, with 80% **3** remaining after 4 days and only 30% after 2 weeks. The NMR spectra do not allow for an unambiguous assignment of the decomposition products.

## Conclusions

Monosilapropofol (**2**) and disilapropofol (**3**) were synthesized via optimized [1,3]-retro-Brook rearrangements in good yields and excellent purities. Comprehensive structural characterization, including single-crystal X-ray diffraction analysis of **2-K**, together with advanced quantum chemical analyses, revealed that carbon–silicon substitution profoundly alters bond polarity and the electrostatic potential while preserving steric similarity. We anticipate that **2** and **3** exhibit enhanced solubility in aqueous media compared to propofol, likely resulting from the increased polarity introduced by the carbon–silicon substitution, while showing slightly reduced lipophilicity. Aqueous stability studies underscored the impact of silicon substitution: monosilapropofol (**2**) undergoes gradual hydrolysis under physiological conditions, whereas disilapropofol (**3**) remains stable in neutral saline solution for weeks. Collectively, these findings highlight carbon–silicon isosterism as a powerful design principle in anesthetic chemistry and provide a foundation for the future development of silaprodrugs and silicon-based bioisosteres. Ongoing studies will address the pharmacological consequences of the carbon–silicon switch in propofol analogues, including receptor binding investigations. Importantly, the silicon analogues are not intended primarily to produce a more potent drug than propofol, but rather to serve as versatile tools for probing its mechanism of action, investigating ligand–receptor interactions, and studying structure–activity relationships.

## Supplementary Material



## Notes

Synthetic procedures, NMR spectra, quantum chemical details, X-ray crystallographic data (PDF)
