# Supplementary material for: Silapropofol: Carbon–Silicon Isosterism in a Key Anesthetic Scaffold
Source: ACS Omega. 2026 Jan 7;11(2):3529–34. doi: 10.1021/acsomega.5c11217 (PMC12824937; doi:10.1021/acsomega.5c11217)
Supplement: Supplementary file 1 [file ao5c11217_si_001.pdf]

Supporting Information

Silapropofol: Carbon–Silicon Isosterism in a Key Anesthetic Scaffold

Sarah Koschabek,[a] Florian Kleemiss,[b] Noel Angel Espinosa-Jalapa,[a] and Jonathan O. Bauer*[a]

[a] Faculty of Chemistry and Pharmacy, Institute of Inorganic Chemistry, University of Regensburg, Universitätsstraße 31, D-93053 Regensburg, Germany

[b] Department of Chemistry, Institute of Inorganic Chemistry, RWTH Aachen University, Landoltweg 1a, D-52074 Aachen, Germany

Corresponding author: jonathan.bauer@ur.de

Table of contents

1. General remarks ... 2
2. Synthetic procedures ... 3
2.1. Synthesis of 2-bromo-6-*iso*-propylphenol (**5**) ... 3
2.2. Synthesis of (2-bromo-6-*iso*-propylphenoxy)dimethylsilane (**6**) ... 4
2.3. Synthesis of 2-(dimethylsilyl)-6-*iso*-propylphenol (monosilapropofol, **2**) ... 6
2.4. Synthesis of potassium 2-(dimethylsilyl)-6-*iso*-propylphenolate (**2-K**) ... 8
2.5. Stability of monosilapropofol (**2**) in aqueous solution ... 8
2.6. Synthesis of (2,6-dibromophenoxy)dimethylsilane (**8**) ... 10
2.7. Synthesis of 2-bromo-6-(dimethylsilyl)phenol (**9**) ... 12
2.8. Synthesis of (2-bromo-6-(dimethylsilyl)phenoxy)dimethylsilane (**10**) ... 14
2.9. Synthesis of 2,6-bis(dimethylsilyl)phenol (disilapropofol, **3**) ... 16
2.10. Stability of disilapropofol (**3**) in aqueous solution ... 18
3. Crystallographic analysis ... 19
4. Quantum chemical calculations ... 21
5. References ... 22

1. General remarks

All experiments were performed under an atmosphere of purified nitrogen using standard Schlenk techniques or in an MBraun Unilab 1200/780 glovebox. Traces of moisture and oxygen were removed from the inert gas by passage through BASF R 3-1 ($CuO/MgSiO_3$), concentrated sulfuric acid, orange gel, and a P_4O_{10}/pumice granulate. Glassware was dried at 140 °C prior to use. Solvents (dichloromethane, diethyl ether, *n*-hexane, *n*-pentane, tetrahydrofuran) were dried and degassed with an MBraun SP800 system and stored over 3 Å molecular sieves. Triethylamine (≥99%, Merck KGaA) was heated at reflux over CaH_2 and distilled prior to use. *n*-Butyllithium (2.5 M in hexanes, Merck KGaA), *tert*-butyllithium (1.6 M in pentane, Merck KGaA), chlorodimethylsilane (99%, Merck KGaA), 2-*iso*-propylphenol (98%, Merck KGaA), *N*-bromosuccinimide (99%, Merck KGaA), di-*iso*-propylamine (>99.5%, Merck KGaA), potassium *tert*-butoxide (≥98%, Merck KGaA), and 2,6-dibromophenol (99%, Merck KGaA) were used as received. C_6D_6 (≥99 %, Merck KGaA) and $CDCl_3$ were dried over 3 Å molecular sieves. NMR spectra were recorded on a Bruker Avance 400 spectrometer (^{1}H NMR: 400.13 MHz, ^{13}C NMR: 100.6 MHz, ^{29}Si NMR: 79.5 MHz, T = 298 K). Chemical shifts (δ) are reported in parts per million (ppm). ^{1}H and ^{13}C{^{1}H} NMR spectra are referenced to tetramethylsilane ($SiMe_4$, δ = 0.0 ppm) as external standard, with the deuterium signal of the solvent serving as internal lock and the residual solvent signal as an additional reference. ^{29}Si{^{1}H} NMR spectra are referenced to $SiMe_4$ (δ = 0.0 ppm). Coupling constants *J* are given in Hertz [Hz]. Signal multiplicities are abbreviated as follows: s = singlet, d = doublet, t = triplet, dd = doublet of doublets, sept = septet. Elemental analyses were performed on a Vario MICRO cube apparatus. High-resolution mass spectrometry was carried out on a Jeol AccuTOF GCX and an Agilent Q-TOF 6540 UHD spectrometer.

2. Synthetic procedures

2.1. Synthesis of 2-bromo-6-*iso*-propylphenol (5)

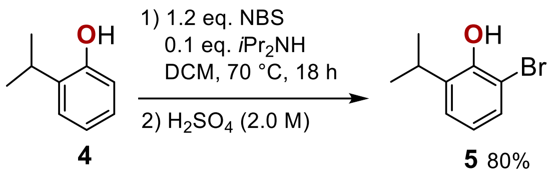

Scheme S1. Synthesis of 2-bromo-6-*iso*-propylphenol (**5**).

The synthesis was performed according to a modified literature procedure.[23]
A 500 mL Schlenk flask equipped with a Soxhlet apparatus was charged with 2-*iso*-propylphenol (9.9 mL, 73.4 mmol, 1.0 equiv.) and di-*iso*-propylamine (1.0 mL, 7.34 mmol, 0.1 equiv.) dissolved in dichloromethane (200 mL). The Soxhlet thimble was filled with *N*-bromosuccinimide (NBS, 15.7 g, 88.1 mmol, 1.2 equiv.). The mixture was refluxed for 18 h under argon, during which the NBS was gradually consumed. After cooling to room temperature, the solution was poured into 2 M sulfuric acid (200 mL). The layers were separated, and the aqueous layer was extracted with DCM (3 × 50 mL). The combined organic extracts were dried over $MgSO_4$, filtered, and concentrated under reduced pressure. The crude residue was purified by Kugelrohr distillation (60 °C, 4.5 × 10^{-3} mbar). The main fraction contained predominantly 2-bromo-6-*iso*-propylphenol (**5**) with minor amounts of unreacted 2-*iso*-propylphenol (6%). The product was obtained as a pale-yellow liquid (12.6 g, 58.7 mmol, 80%).

^{1}H NMR (400.1 MHz, C_6D_6): δ = 1.22 (d, 6H, $^3J_{HH}$ = 7.0 Hz, C*H*$_3$), 3.31 (sept, 1H, $^3J_{HH}$ = 7.0 Hz, C*H*(CH$_3$)$_2$), 5.38 (s, 1H, O*H*), 6.47 (t, 1H, $^3J_{HH}$ = 7.9 Hz, *p-H*), 6.90 (dd, 1H, $^4J_{HH}$ = 1.2 Hz, $^3J_{HH}$ = 7.8 Hz, *m-H*), 7.07 (dd, 1H, $^4J_{HH}$ = 1.4 Hz, $^3J_{HH}$ = 8.0 Hz, *m-H*). **^{13}C{^{1}H} NMR** (100.6 MHz, $CDCl_3$): δ = 22.4 (*C*H$_3$), 28.0 (*C*H(CH$_3$)$_2$), 110.6 (*o-C*$_{Ar}$), 121.5 (*p-C*$_{Ar}$), 126.0 (*m-C*$_{Ar}$), 129.1 (*m-C*$_{Ar}$), 136.4 (*o-C*$_{Ar}$), 149.4 (*i-C*$_{Ar}$).

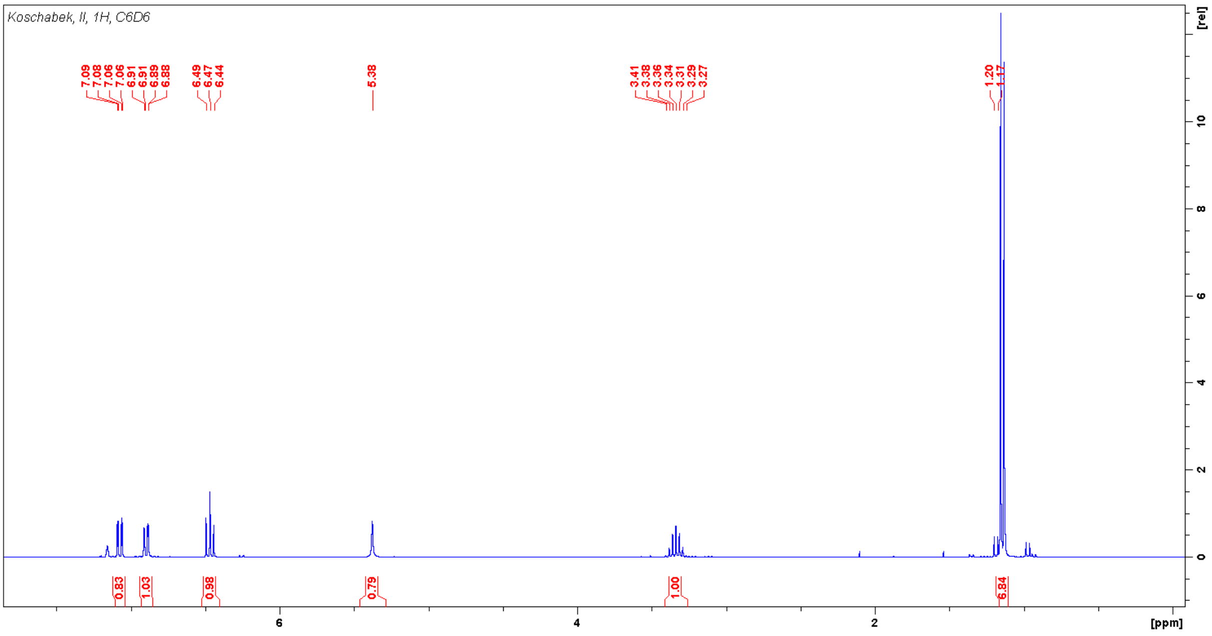

Figure S2. ^{1}H NMR spectrum (C_6D_6) of compound **5**.

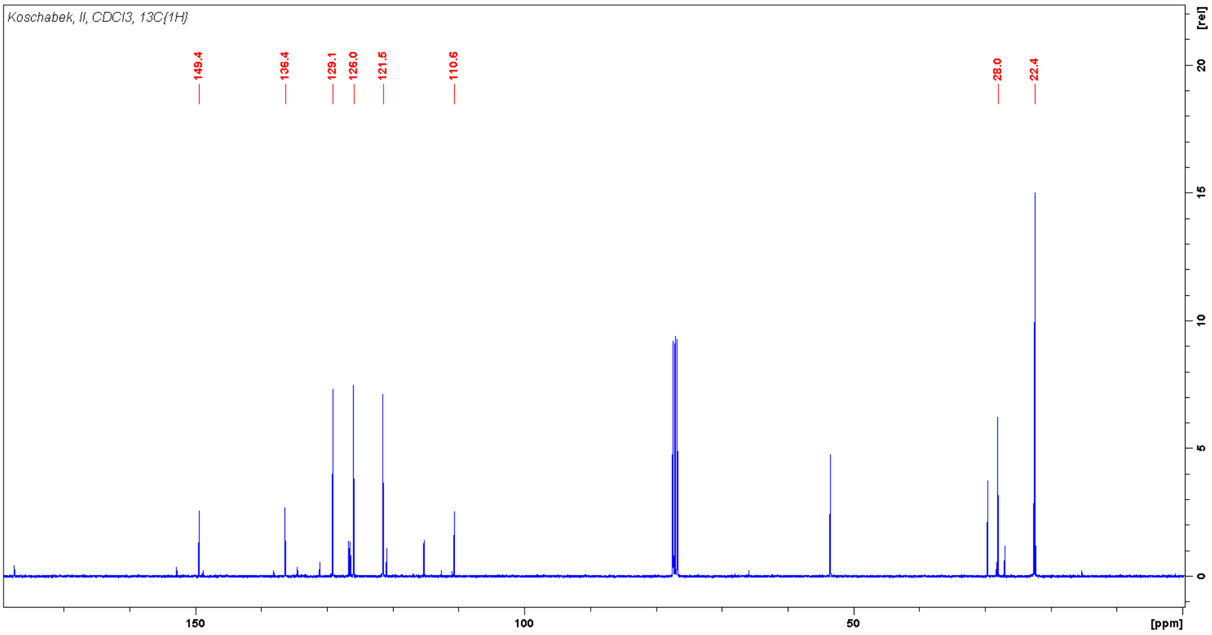

Figure S3. $^{13}C\{^1H\}$ NMR spectrum ($CDCl_3$) of compound **5**.

2.2. Synthesis of (2-bromo-6-*iso*-propylphenoxy)dimethylsilane (6)

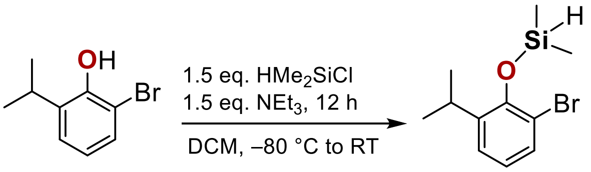

5 **6** 93%

Scheme S2. Synthesis of (2-bromo-6-*iso*-propylphenoxy)dimethylsilane (**6**).

To a 1 L Schlenk flask equipped with a magnetic stir bar, 2-bromo-6-*iso*-propylphenol (**5**, 33.4 g, 155 mmol, 1.0 equiv.) was dissolved in dry dichloromethane (500 mL) and cooled to –80 °C. Chlorodimethylsilane (26.0 mL, 233 mmol, 1.5 equiv.) was added via syringe, followed by slow addition of triethylamine (32.3 mL, 233 mmol, 1.5 equiv). The reaction mixture was allowed to warm gradually to room temperature and stirred for 12 h, resulting in a white suspension. Volatiles were removed under reduced pressure, and the residue was suspended in pentane (150 mL). The suspension was filtered through a Celite pad and washed with additional pentane (3 × 25 mL). The combined filtrates were concentrated, and the crude orange liquid was purified by Kugelrohr distillation. The main fraction contained (2-bromo-6-*iso*-propylphenoxy)dimethylsilane (**6**) as a pale-yellow liquid (30.9 g, 113 mmol, 93%).

^{1}H NMR (400.1 MHz, C_6D_6): δ = 0.27 (d, 6H, $^3J_{HH}$ = 2.9 Hz, Si(C*H*$_3$)$_2$), 1.08 (d, 6H, $^3J_{HH}$ = 7.0 Hz, C*H*$_3$), 3.31 (sept, 1H, $^3J_{HH}$ = 6.9 Hz, C*H*(CH$_3$)$_2$), 5.20 (sept, 1H, $^3J_{HH}$ 2.9 Hz, Si*H*), 6.57 (t, 1H, $^3J_{HH}$ = 7.8 Hz, *p-H*), 6.93 (dd, 1H, $^4J_{HH}$ = 1.5 Hz, $^3J_{HH}$ = 7.8 Hz, *m-H*), 7.28 (dd, 1H, $^4J_{HH}$ = 1.5 Hz, $^3J_{HH}$ = 7.9 Hz, *m-H*). **^{13}C{^{1}H} NMR** (100.6 MHz, C_6D_6): δ = -0.8 (Si(*C*H$_3$)$_2$), 23.2 (*C*H$_3$), 27.8 (*C*H), 116.1 (*o-C*$_{Ar}$), 121.5 (*p-C*$_{Ar}$), 123.7 (*m-C*$_{Ar}$), 125.6 (*m-C*$_{Ar}$), 130.8 (*o-C*$_{Ar}$), 150.3 (*i-C*$_{Ar}$). **^{29}Si NMR** (79.5 MHz, C_6D_6): δ = 6.93 (dsept, $^2J_{SiH}$ = 7.0 Hz, $^1J_{SiH}$ = 212.6 Hz). **HRMS** (EI+): calcd. m/z for $C_{11}H_{17}BrOSi$ [M$^+$]: 272.02266; found: 272.02205. **EA**: calcd. for $C_{11}H_{17}BrOSi$: C 48.35, H 6.27; found: C 48.14, H 6.04.

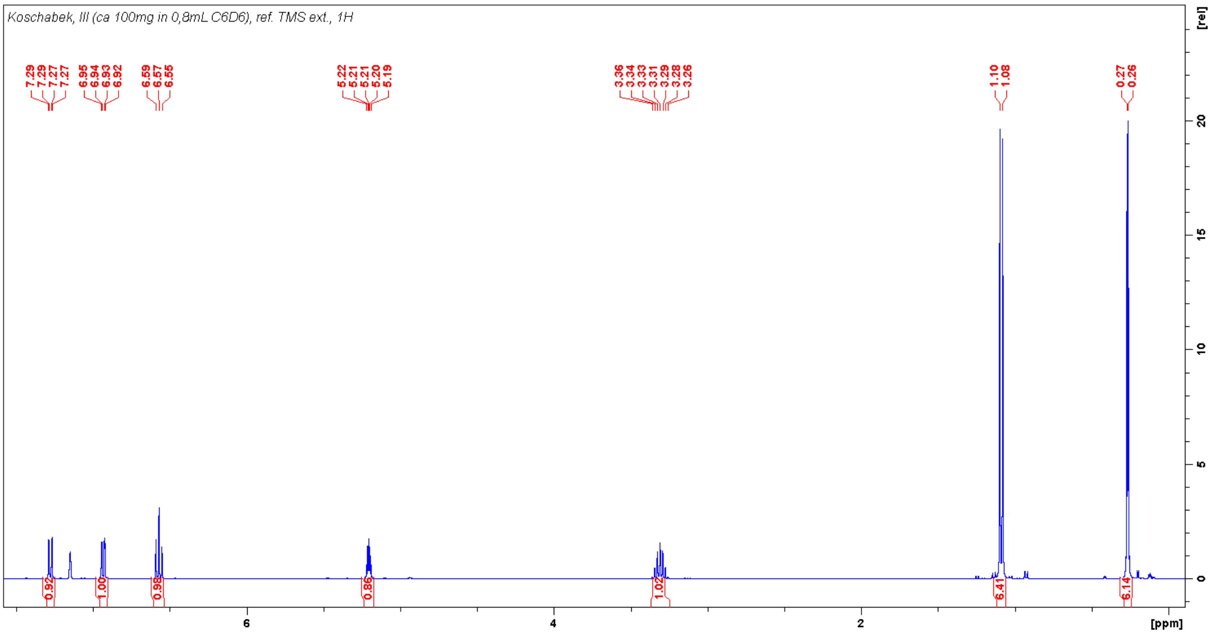

Figure S3. ^{1}H NMR spectrum (C_6D_6) of compound **6**.

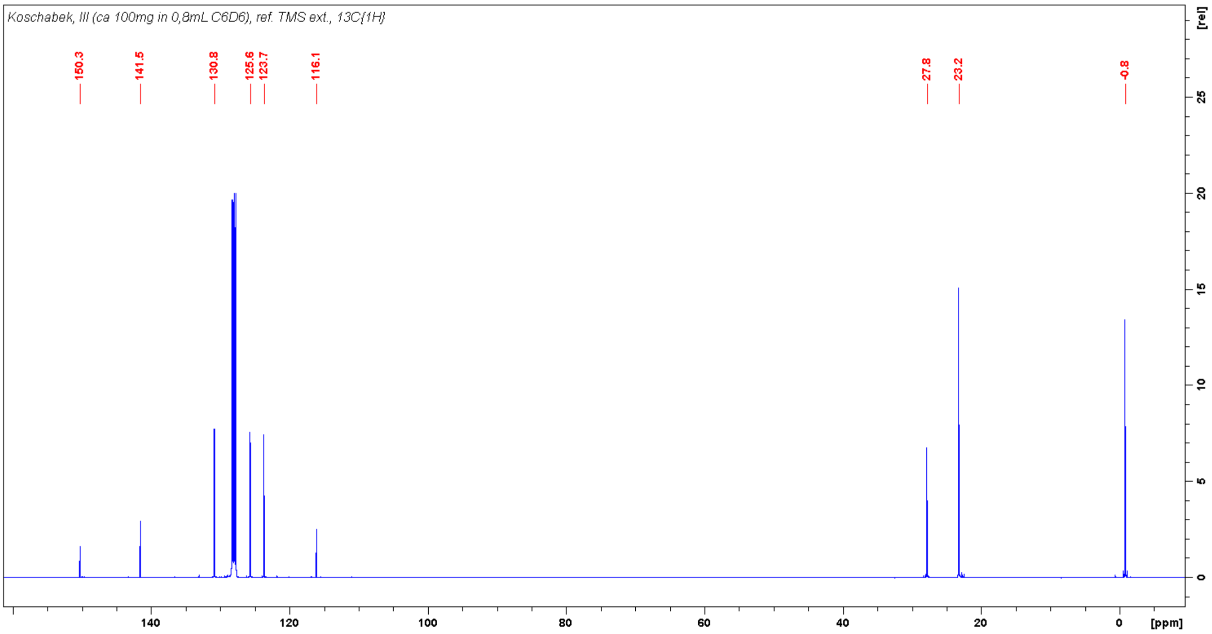

Figure S4. $^{13}C\{^1H\}$ NMR spectrum (C_6D_6) of compound **6**.

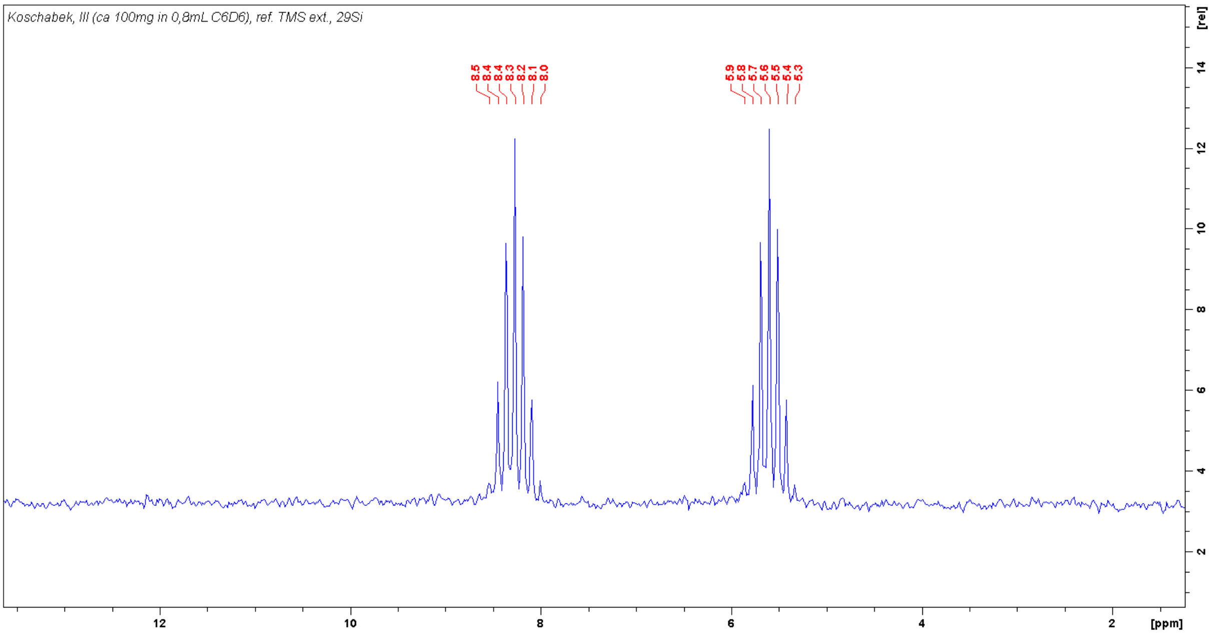

Figure S5. ^{29}Si NMR spectrum (C_6D_6) of compound **6**.

2.3. Synthesis of 2-(dimethylsilyl)-6-*iso*-propylphenol (monosilapropofol, 2)

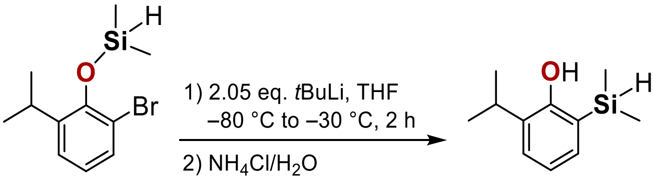

6 **2** 55%

Scheme S3. Synthesis of 2-(dimethylsilyl)-6-*iso*-propylphenol (monosilapropofol, **2**).

Table S1. Optimization of reaction conditions for the synthesis of monosilapropofol (**2**).

**Entry**	**Solvent**	**Reagent**	**Temperature, Time**	**Yield (determined by ^{1}H NMR)**
a	THF	*n*BuLi (1.0 equiv.)	–80 °C to –30 °C, 2 h	56%
b	THF	*n*BuLi (1.0 equiv.)	–80 °C to RT, 24 h	44%
c	Et_2O	*n*BuLi (1.0 equiv.)	–80 °C to –30 °C, 2 h	0%
d	THF	*n*BuLi (1.1 equiv.)	–80 °C to –30 °C, 2 h	60%
e	THF	*t*BuLi (2.05 equiv.)	–80 °C to –30 °C, 2 h	92%

A Schlenk flask was charged with compound **6** (2.82 g, 10.3 mmol, 1.0 equiv.) in dry tetrahydrofuran (50 mL) and cooled to –80 °C (Table S1, Entry e). *tert*-Butyllithium (13.2 mL, 21.2 mmol, 2.05 equiv., 1.6 M in pentane) was added dropwise via syringe, resulting in a yellow solution. After 2 h, the mixture was allowed to warm slowly to –30 °C and was then quenched with saturated aqueous NH_4Cl (50 mL). The layers were separated, and the aqueous phase was extracted with diethyl ether (2 × 50 mL). The combined organic layers were dried over $MgSO_4$,

filtered, and concentrated under reduced pressure. The crude product consisted of monosilapropofol (**2**, 92%) and compound **5** (8%) as determined by ^{1}H NMR spectroscopy. The crude product was purified by Kugelrohr distillation (60 °C, 3.2 × 10^{-3} mbar), followed by recrystallization from *n*-hexane at –80 °C, affording monosilapropofol (**2**) as an amorphous solid (1.11 g, 5.66 mmol, 55%).

^{1}H NMR (400.1 MHz, C_6D_6): δ = 0.36 (d, 6H, $^{3}J_{HH}$ = 3.7 Hz, Si(CH_3)$_2$), 1.04 (d, 6H, $^{3}J_{HH}$ = 6.9 Hz, CH_3), 2.64 (sept, 1H, $^{3}J_{HH}$ = 6.8 Hz, C*H*(CH$_3$)$_2$), 4.68 (s, 1H, O*H*), 4.75 (sept, 1H, $^{3}J_{HH}$ 3.7 Hz, Si*H*), 6.91 (t, 1H, $^{3}J_{HH}$ = 7.4 Hz, *p-H*), 7.12 (dd, 1H, $^{4}J_{HH}$ = 1.6 Hz, $^{3}J_{HH}$ = 7.6 Hz, *m-H*), 7.28 (dd, 1H, $^{4}J_{HH}$ = 1.6 Hz, $^{3}J_{HH}$ = 7.1 Hz, *m-H*). **^{13}C{^{1}H} NMR** (100.6 MHz, C_6D_6): δ = –3.5 (Si(*C*H$_3$)$_2$), 22.8 (*C*H$_3$), 27.1 (*C*H), 121.5 (*o*-C_{Ar}), 122.7 (*p*-C_{Ar}), 128.1 (*m*-C_{Ar}), 132.8 (*m*-C_{Ar}), 133.5 (*o*-C_{Ar}), 158.0 (*i*-C_{Ar}). **^{29}Si{^{1}H} NMR** (79.5 MHz, C_6D_6): δ = –21.20 (s). **HRMS** (EI+): calcd. m/z for $C_{11}H_{18}OSi$ [M$^+$]: 194.11214; found: 194.11252. **EA**: calcd. for $C_{11}H_{18}OSi$: C 67.98, H 9.34; found: C 66.94, H 9.25.

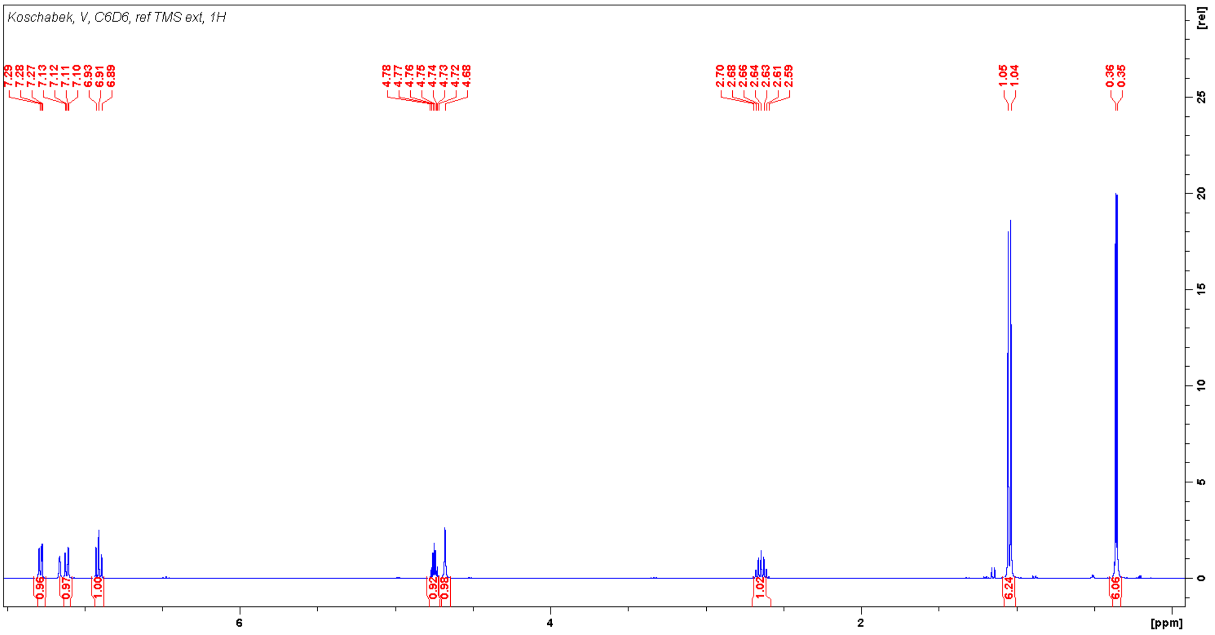

Figure S6. ^{1}H NMR spectrum (C_6D_6) of compound **2**.

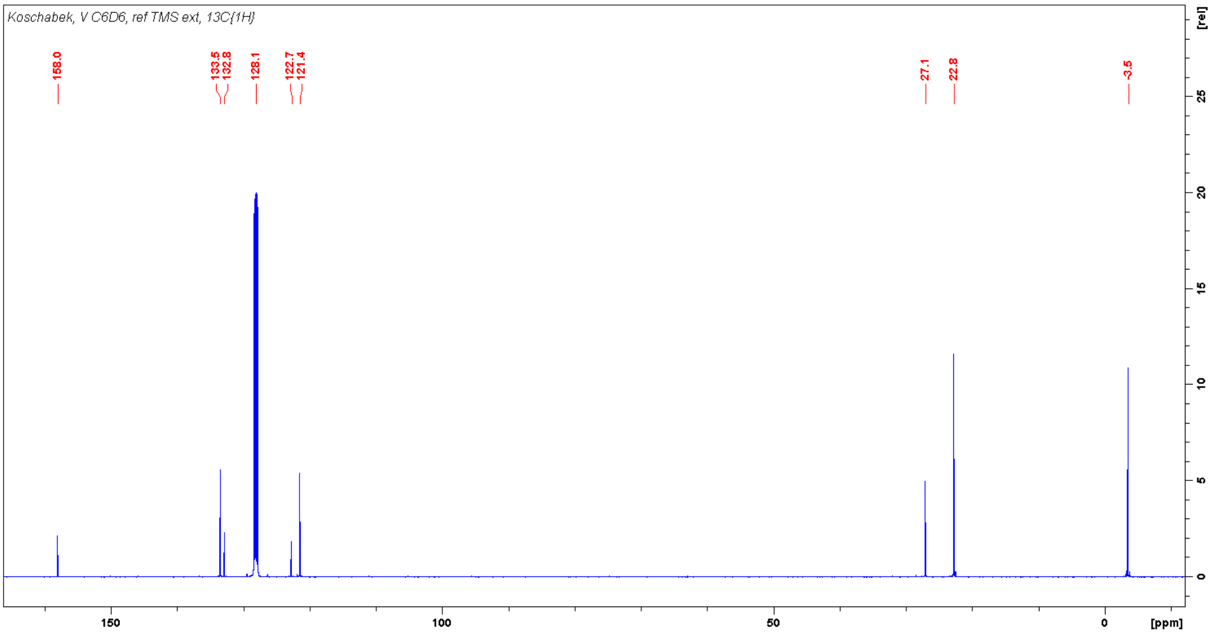

Figure S7. ^{13}C{^{1}H} NMR spectrum (C_6D_6) of compound **2**.

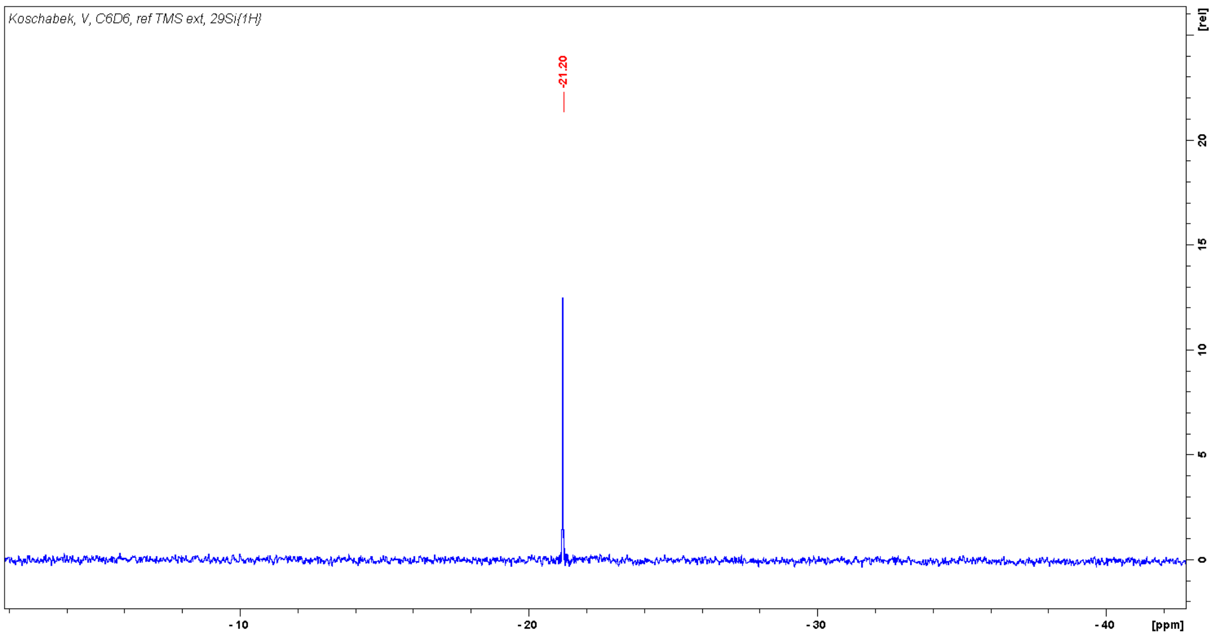

Figure S8. $^{29}Si\{^1H\}$ NMR spectrum (C_6D_6) of compound **2**.

2.4. Synthesis of potassium 2-(dimethylsilyl)-6-*iso*-propylphenolate (2-K)

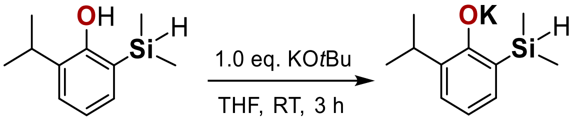

Scheme S4. Synthesis of potassium 2-(dimethylsilyl)-6-*iso*-propylphenolate (**2-K**).

In a 50 mL Schlenk flask, monosilapropofol (**2**, 610 mg, 3.14 mmol, 1.0 equiv.) was dissolved in dry tetrahydrofuran (20 mL) under argon. Potassium *tert*-butoxide (352 mg, 3.14 mmol, 1.0 equiv.) was added in one portion, and the mixture was stirred at room temperature for 3 h. The solvent was removed under reduced pressure, and the residue was washed with diethyl ether (2 × 10 mL) to give potassium 2-(dimethylsilyl)-6-*iso*-propylphenolate (**2-K**) as a pale-yellow solid. Colorless needles suitable for single-crystal X-ray diffraction analysis were obtained by slow evaporation of a tetrahydrofuran solution.

2.5. Stability of monosilapropofol (2) in aqueous solution

The stability of monosilapropofol (**2**) was investigated in aqueous media by NMR spectroscopy. In 0.9% NaCl/D_2O at room temperature, **2** gradually decomposed from 98% at t = 0 to 92% after 1 week, 78% after 2 weeks, and was nearly completely consumed after 6 weeks (Figure S9). In 10 mM $NaHCO_3$/D_2O (pH ≈ 8), decomposition proceeded more rapidly, with only 60% of **2** remaining after 4 days and significant line broadening after 1 week (Figure S10). Based on the NMR spectra, an exact assignment of the decomposition products cannot be made. However, the still observable Si–H signal indicate that dimethylsilanol is likely formed as one of the hydrolysis products, together with 2-*iso*-propylphenol and other unidentified side products.

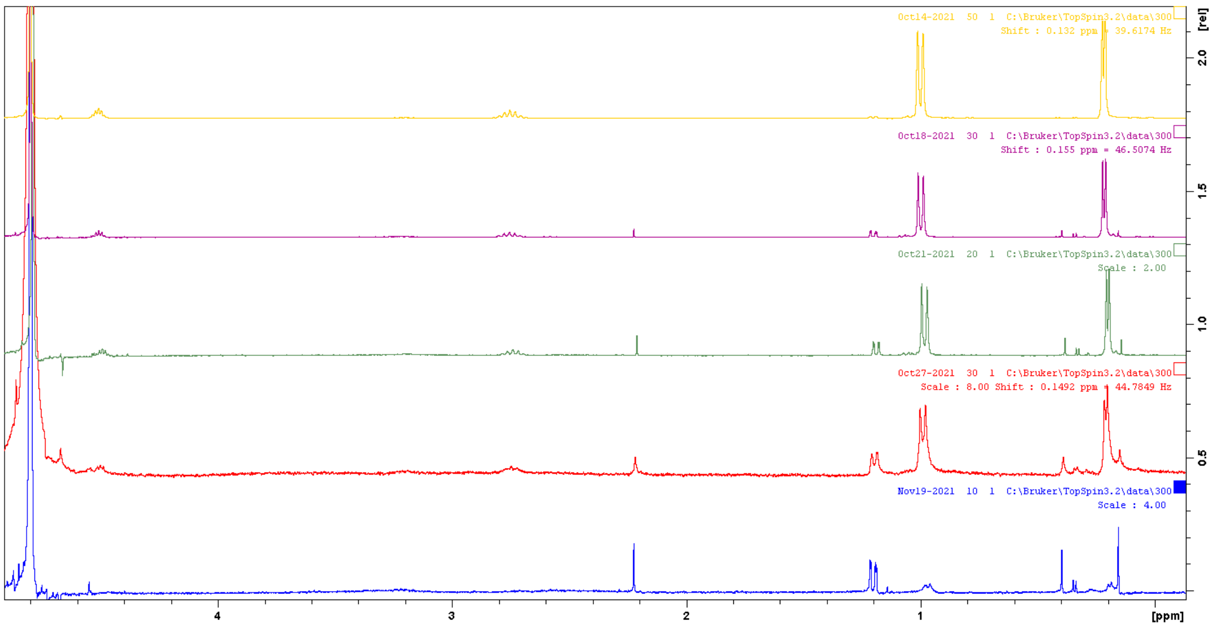

Figure S9. Stability of monosilapropofol (**2**) monitored by ^{1}H NMR spectroscopy in D_2O containing 0.9% NaCl at room temperature. Spectra recorded on the same day (yellow), after 4 days (purple), 7 days (green), 13 days (red), and 44 days (blue).

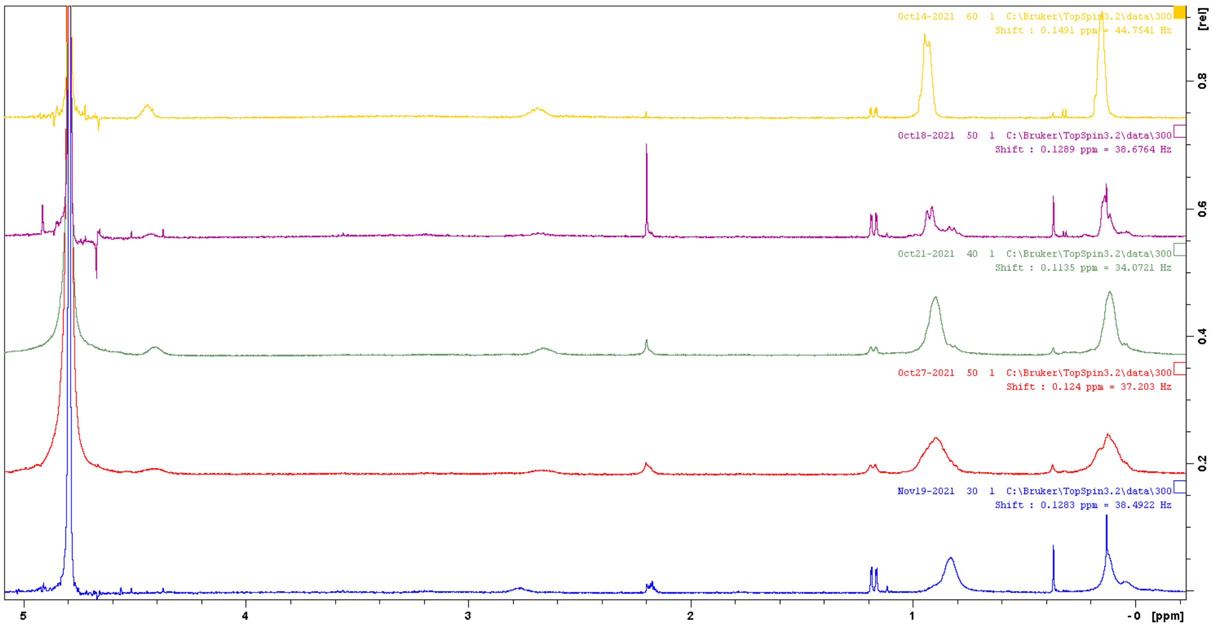

Figure S10. Stability of monosilapropofol (**2**) monitored by ^{1}H NMR spectroscopy in D_2O containing 10 mM $NaHCO_3$ (pH ≈ 8) at room temperature. Spectra recorded on the same day (yellow), after 4 days (purple), 7 days (green), 13 days (red), and 44 days (blue).

2.6. Synthesis of (2,6-dibromophenoxy)dimethylsilane (8)

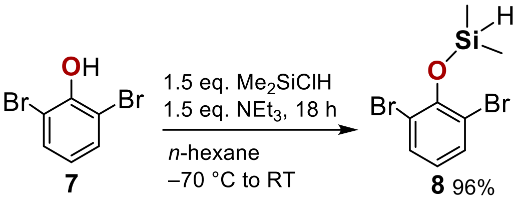

Scheme S5. Synthesis of (2,6-dibromophenoxy)dimethylsilane (**8**).

In a 500 mL Schlenk flask equipped with a magnetic stir bar, 2,6-dibromophenol (**7**, 10.00 g, 39.70 mmol, 1.0 equiv.) was dissolved in dry *n*-hexane (150 mL) and cooled to −70 °C. Chlorodimethylsilane (6.6 mL, 59.55 mmol, 1.5 equiv.) was added slowly via syringe, followed by the dropwise addition of triethylamine (8.3 mL, 59.55 mmol, 1.5 equiv.). The mixture was allowed to warm gradually to room temperature and stirred under argon for 18 h, forming a white suspension. The mixture was filtered through a Celite pad into a 500 mL Schlenk flask, and the filter cake was extracted with *n*-hexane (2 × 50 mL). The combined filtrates were concentrated under reduced pressure to afford (2,6-dibromophenoxy)dimethylsilane (**8**) as a colorless liquid (11.77 g, 37.96 mmol, 96%).

^{1}H NMR (400.1 MHz, C_6D_6): δ = 0.30 (d, 6H, $^3J_{HH}$ = 2.9 Hz, C*H*3), 5.27 (sept, 1H, $^3J_{HH}$ = 2.9 Hz, Si*H*(CH_3)2), 6.12 (t, 1H, $^3J_{HH}$ = 8.0 Hz, *p-H*), 7.12 (d, 2H, $^3J_{HH}$ = 8.0 Hz, *m-H*). **^{13}C{^{1}H} NMR** (100.6 MHz, C_6D_6): δ = –0.7 (*C*H3), 116.5 (*o*-C_{Ar}), 124.1 (*p*-C_{Ar}), 132.6 (*m*-C_{Ar}), 150.9 (*i*-C_{Ar}). **^{29}Si{^{1}H} NMR** (79.5 MHz, C_6D_6): δ = 9.38 (s). **HRMS** (EI+): calcd. m/z for $C_8H_{10}Br_2OSi$ [M$^+$]: 307.88622; found: 307.88666. **EA**: calcd. for $C_8H_{10}Br_2OSi$: C 30.99, H 3.25; found: C 30.80, H 3.14.

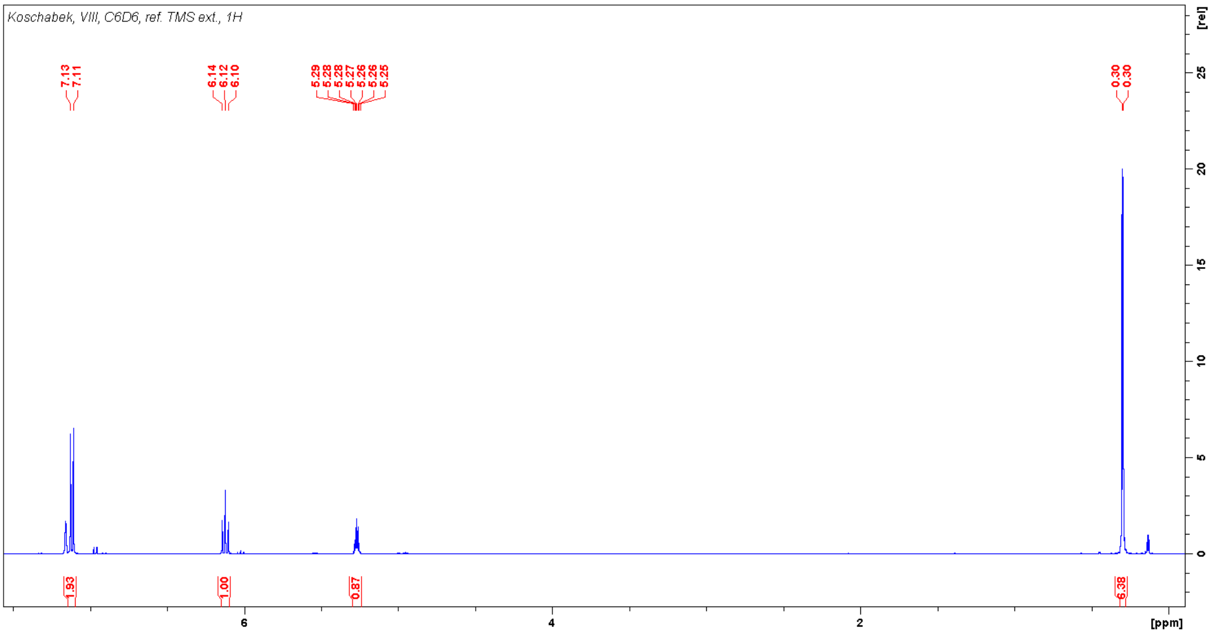

Figure S11. ^{1}H NMR spectrum (C_6D_6) of compound **8**.

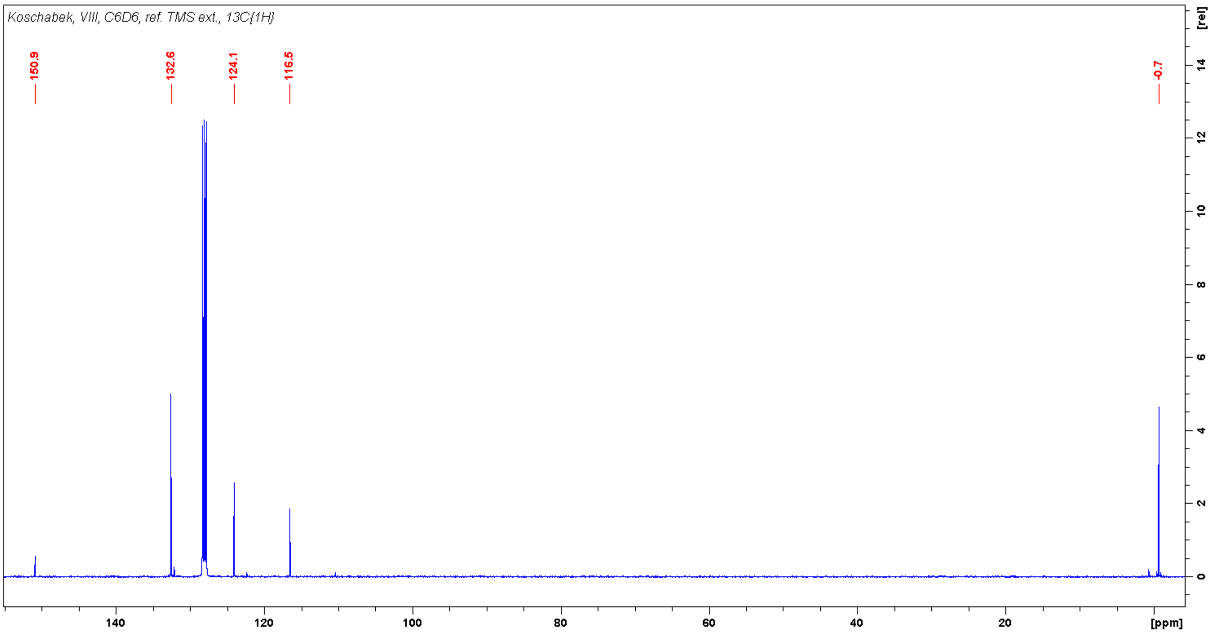

Figure S12. $^{13}C\{^1H\}$ NMR spectrum (C_6D_6) of compound **8**.

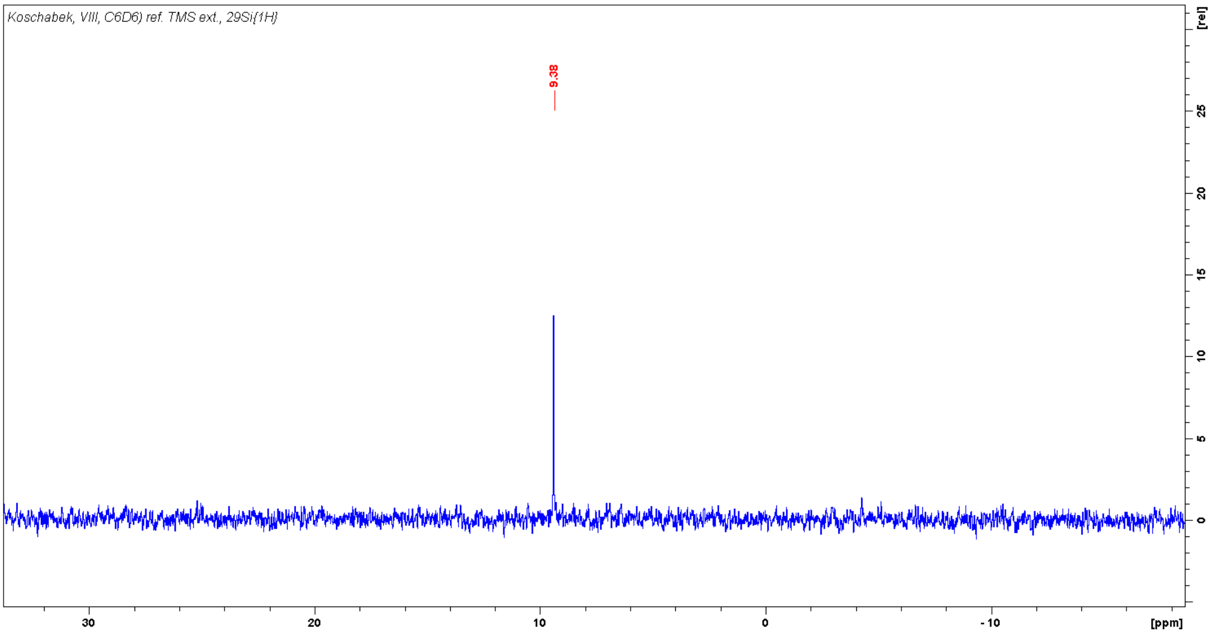

Figure S13. $^{29}Si\{^1H\}$ NMR spectrum (C_6D_6) of compound **8**.

2.7. Synthesis of 2-bromo-6-(dimethylsilyl)phenol (9)

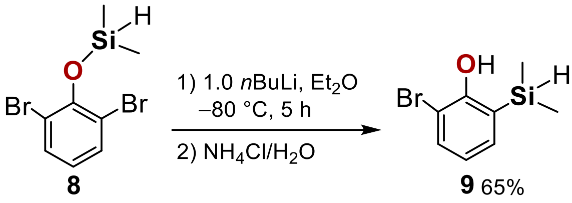

Scheme S6. Synthesis of 2-bromo-6-(dimethylsilyl)phenol (**9**).

Table S2. Optimization of reaction conditions for the synthesis of compound **9**.

**Entry**	**Solvent**	**Reagent**	**Temperature, Time**	**Yield (determined by ^{1}H NMR)**
a	Et_2O	*t*BuLi (2.0 equiv.)	−80 °C to −30 °C, 2 h	66%
b	THF	*t*BuLi (2.0 equiv.)	−80 °C to −30 °C, 2 h	25%
c	Et_2O	*n*BuLi (1.0 equiv.)	−80 °C, 5 h	82%
d	THF	*n*BuLi (1.0 equiv.)	−80 °C to RT, 24 h	36%

In a 500 mL Schlenk flask, compound **8** (11.39 g, 36.72 mmol, 1.0 equiv.) was dissolved in dry diethyl ether (350 mL) and cooled to −80 °C (Table S2, Entry c). A solution of *n*-butyllithium (14.7 mL, 36.72 mmol, 1.0 equiv., 2.5 M in hexanes) was added dropwise via syringe, causing the solution to turn pale yellow. The reaction was stirred for 5 h at −80 °C and quenched with saturated aqueous NH_4Cl (200 mL). The phases were separated, and the aqueous layer was extracted with diethyl ether (2 × 200 mL). The combined organic extracts were dried over $MgSO_4$, filtered, and concentrated. The crude product consisted of 2-bromo-6-(dimethylsilyl)phenol (**9**, 82%) and 2,6-dibromophenol (**7**, 18%) as determined by ^{1}H NMR spectroscopy. Kugelrohr distillation (40 °C, 8.1 × 10^{-3} mbar) afforded a slightly enriched mixture of compound **9** (86 %) and **7** (14 %). Subsequent recrystallization from *n*-hexane at −80 °C provided colorless needles of compound **7**[45] and 2-bromo-6-(dimethylsilyl)phenol (**9**) as a colorless liquid (7.61 g, 23.90 mmol, 65 %), which was obtained after removal of the solvent under reduced pressure.

^{1}H NMR (400.1 MHz, C_6D_6): δ = 0.34 (d, 6H, $^3J_{HH}$ = 3.7 Hz, C*H*$_3$), 4.69 (sept, 1H, $^3J_{HH}$ = 3.7 Hz, Si*H*(CH_3)$_2$), 5.42 (s, 1H, O*H*), 6.45 (t, 1H, $^3J_{HH}$ = 7.5 Hz, *p-H*), 7.17 (dd, 1H, $^4J_{HH}$ = 1.6 Hz, $^3J_{HH}$ = 8.1 Hz, *m-H*), 7.21 (dd, 1H, $^4J_{HH}$ = 1.6 Hz, $^3J_{HH}$ = 7.2 Hz, *m-H*). **^{13}C{^{1}H} NMR** (100.6 MHz, C_6D_6): δ = –3.8 (CH_3), 110.6 (*o*-C_{Ar}), 122.1 (*o*-C_{Ar}), 124.9 (*p*-C_{Ar}), 133.7 (*m*-C_{Ar}), 135.7 (*m*-C_{Ar}), 156.7 (*i*-C_{Ar}). **^{29}Si{^{1}H} NMR** (79.5 MHz, C_6D_6): δ = –16.84 (s). **HRMS** (GC-MS): calcd. m/z for $C_8H_{11}BrOSi$ [M^+]: 229.97625; found: 229.96480. **EA**: calcd. for $C_8H_{11}BrOSi$: C 41.57, H 4.80; found: C 41.41, H 4.85.

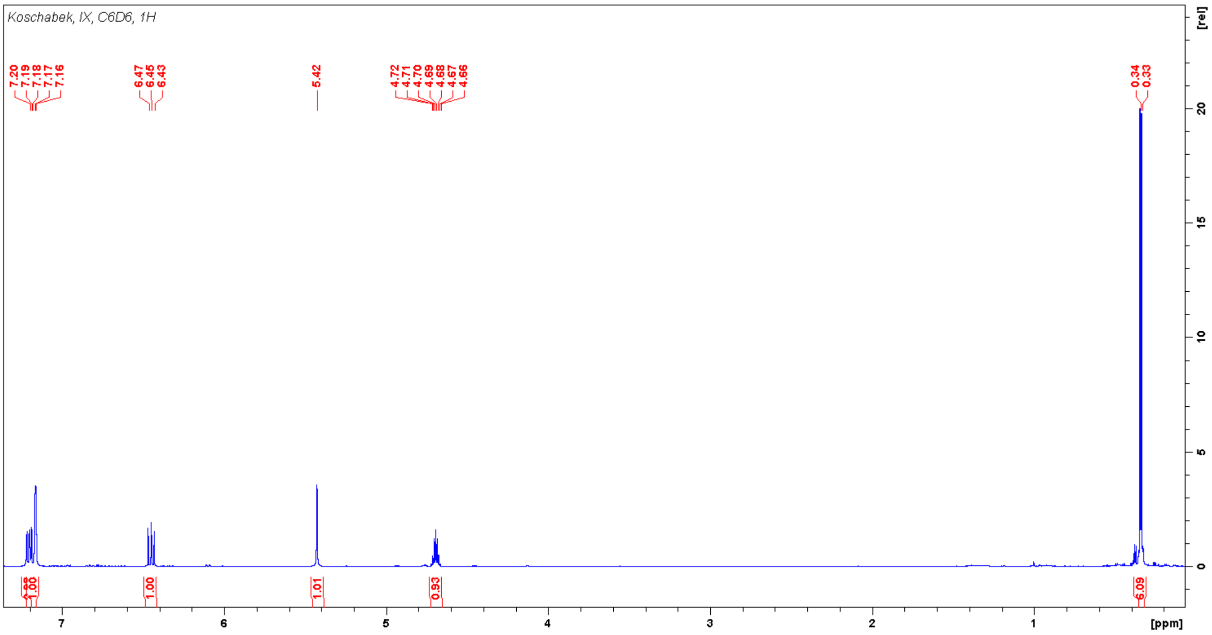

Figure S14. ^{1}H NMR spectrum (C_6D_6) of compound **9**.

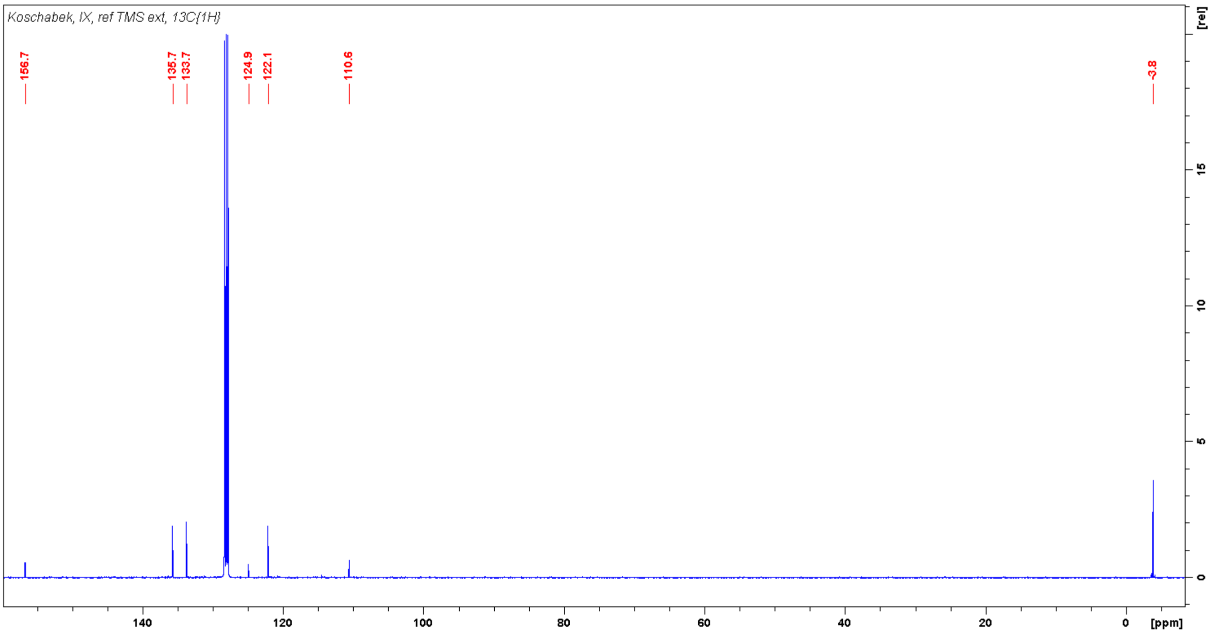

Figure S15. $^{13}C\{^{1}H\}$ NMR spectrum (C_6D_6) of compound **9**.

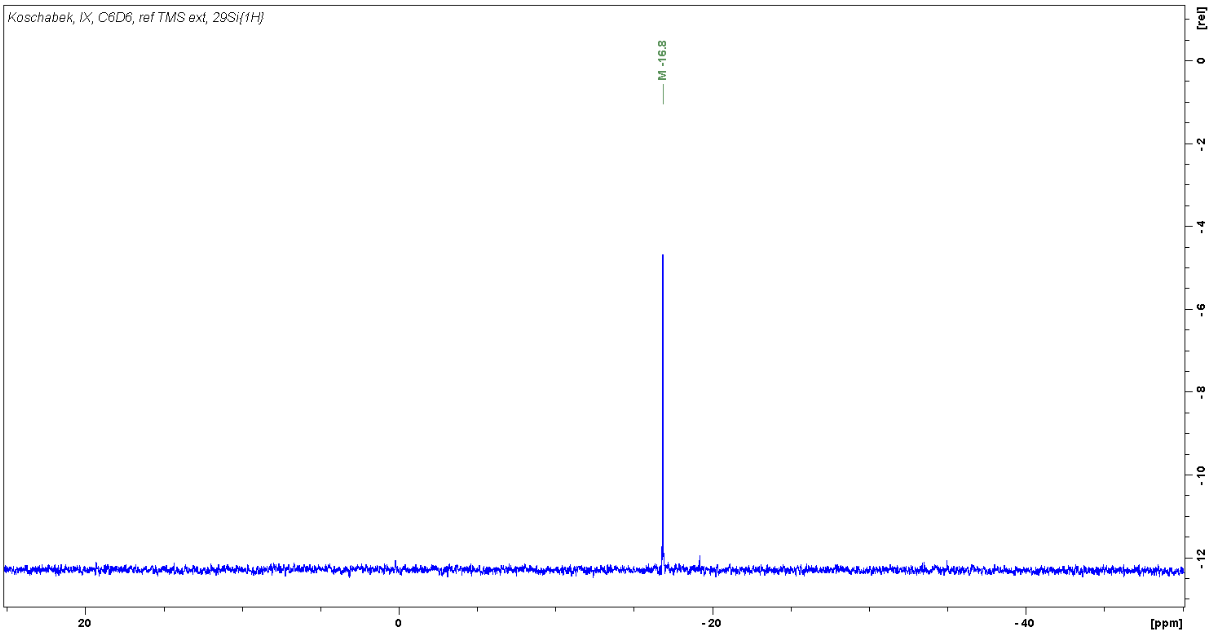

Figure S16. $^{29}Si\{^1H\}$ NMR spectrum (C_6D_6) of compound **9**.

2.8. Synthesis of (2-bromo-6-(dimethylsilyl)phenoxy)dimethylsilane (10)

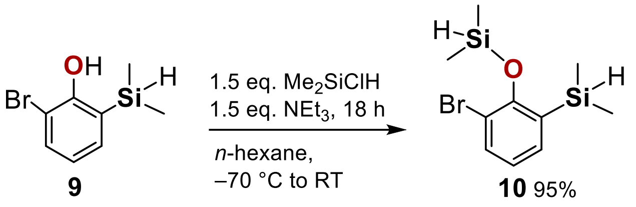

Scheme S7. Synthesis of (2-bromo-6-(dimethylsilyl)phenoxy)dimethylsilane (**10**).

To a 500 mL Schlenk flask containing compound **9** (7.94 g, 34.36 mmol, 1.0 equiv.) in dry *n*-hexane (250 mL) at −70 °C were added chlorodimethylsilane (5.7 mL, 51.54 mmol, 1.5 equiv.) and triethylamine (7.1 mL, 51.54 mmol, 1.5 equiv.) dropwise under argon. The mixture was allowed to warm to room temperature and stirred for 18 h, forming a turbid suspension. After filtration through a Celite pad and washing with *n*-hexane (3 × 25 mL), the filtrate was concentrated to give a crude yellow liquid. Kugelrohr distillation (70 °C, 2.2 × 10^{-3} mbar) afforded (2-bromo-6-(dimethylsilyl)phenoxy)dimethylsilane (**10**) as a colorless liquid (9.41 g, 32.5 mmol, 95%).

^{1}H NMR (400.1 MHz, C_6D_6): δ = 0.25 (d, 6H, $^3J_{HH}$ = 3.8 Hz, OSiCH_3), 0.32 (d, 6H, $^3J_{HH}$ = 2.8 Hz, SiCH_3), 4.68 (sept, 1H, $^3J_{HH}$ = 3.7 Hz, OSiH(CH_3)$_2$), 5.39 (sept, 1H, $^3J_{HH}$ = 2.9 Hz, SiH(CH_3)$_2$), 6.56 (t, 1H, $^3J_{HH}$ = 7.5 Hz, *p-H*), 7.14 (dd, 1H, $^4J_{HH}$ = 1.7 Hz, $^3J_{HH}$ = 7.3 Hz, *m-H*), 7.42 (dd, 1H, $^4J_{HH}$ = 1.6 Hz, $^3J_{HH}$ = 7.9 Hz, *m-H*). **^{13}C{^{1}H} NMR** (100.6 MHz, C_6D_6): δ = –4.0 (SiCH_3), –0.7 (OSiCH_3), 115.3 (*o*-C_{Ar}), 123.7 (*o*-C_{Ar}), 130.9 (*p*-C_{Ar}), 134.8 (*m*-C_{Ar}), 135.4 (*m*-C_{Ar}), 157.3 (*i*-C_{Ar}). **^{29}Si{^{1}H} NMR** (79.5 MHz, C_6D_6): δ = –19.96 (s, *Si*H(CH_3)$_2$), 6.57 (s, O*Si*H(CH_3)$_2$). **HRMS** (EI+): calcd. m/z for $C_{10}H_{17}BrOSi_2$ [M^+]: 287.99958; found: 288.00014. **EA**: calcd. for $C_{10}H_{17}BrOSi_2$: C 41.51, H 5.92; found: C 41.52, H 5.92.

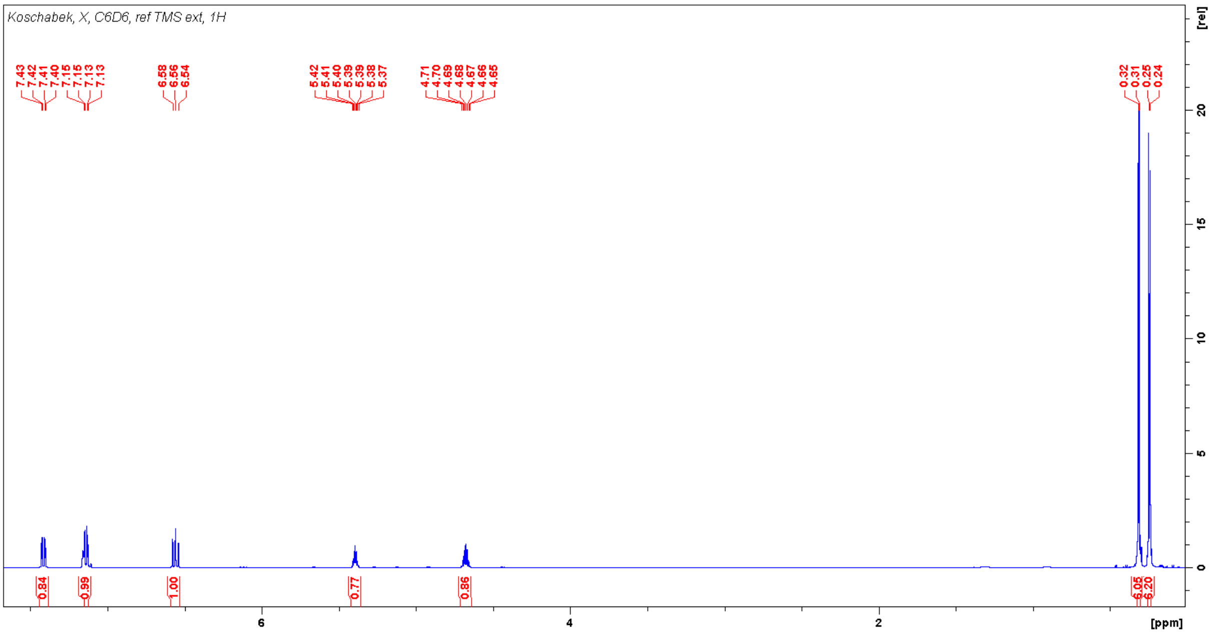

Figure S17. ^{1}H NMR spectrum (C_6D_6) of compound **10**.

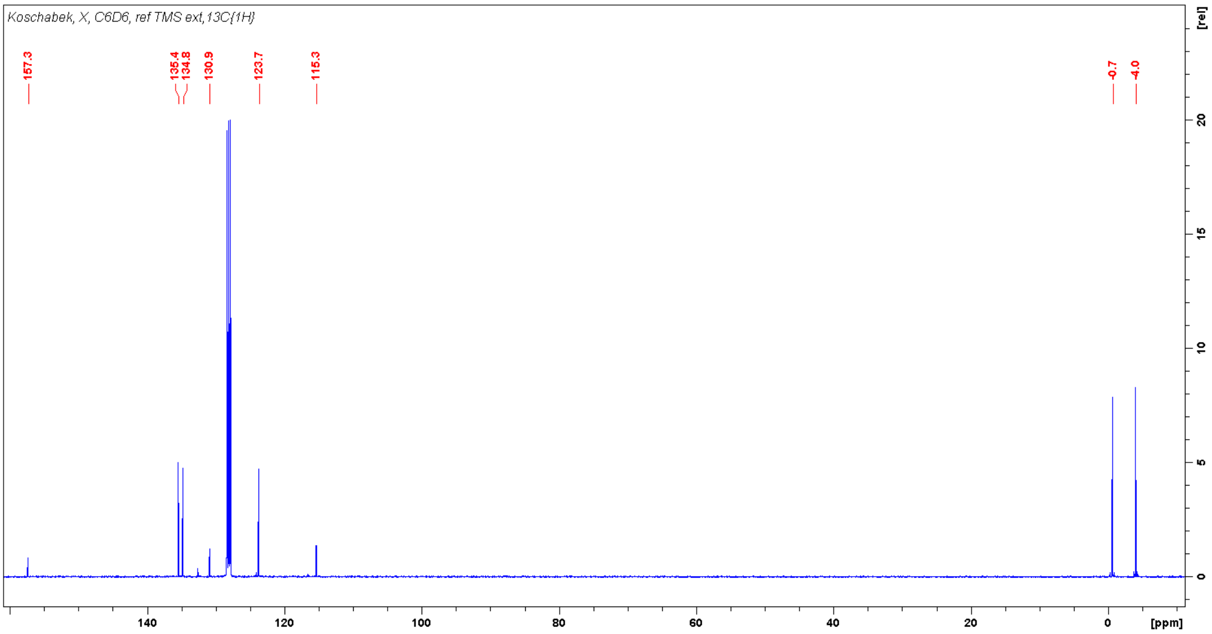

Figure S18. ^{13}C{^{1}H} NMR spectrum (C_6D_6) of compound **10**.

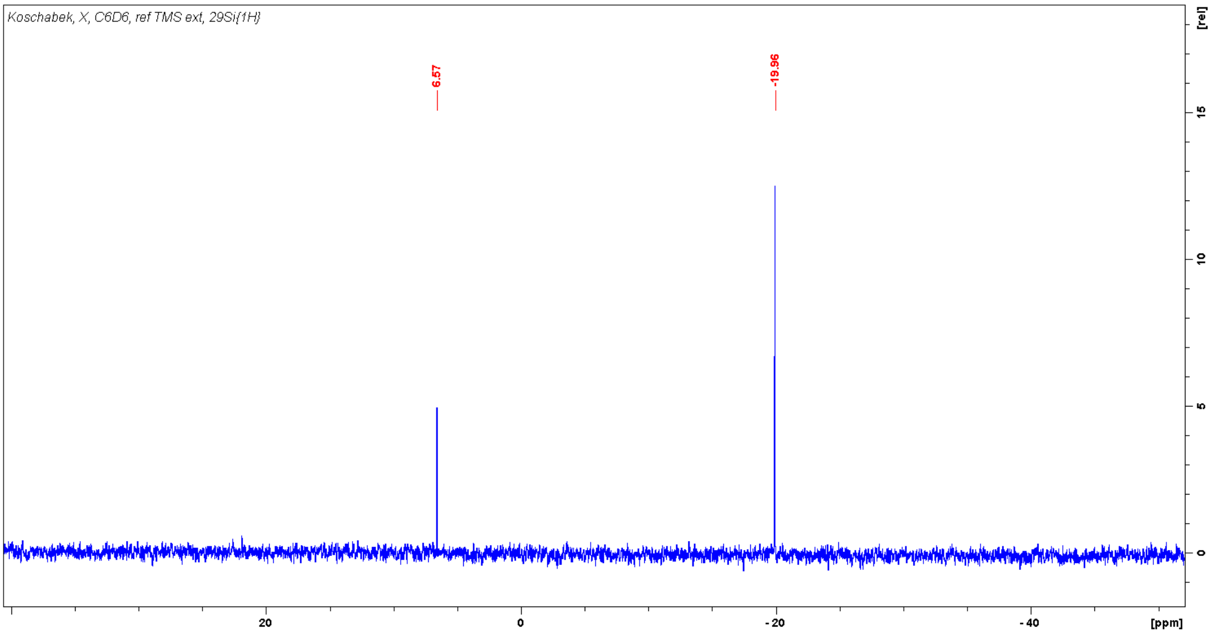

Figure S19. $^{29}Si\{^1H\}$ NMR spectrum (C_6D_6) of compound **10**.

2.9. Synthesis of 2,6-bis(dimethylsilyl)phenol (disilapropofol, 3)

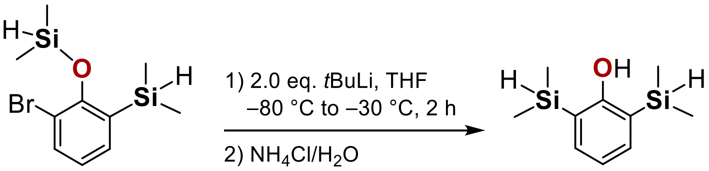

Scheme S8. Synthesis of 2,6-bis(dimethylsilyl)phenol (disilapropofol, **3**).

In a 100 mL Schlenk flask, compound **10** (3.32 g, 11.58 mmol, 1.0 equiv.) was dissolved in dry tetrahydrofuran (50 mL) and cooled to −80 °C. *tert*-Butyllithium (13.6 mL, 23.16 mmol, 2.0 equiv., 1.7 M in pentane) was added slowly via syringe. The mixture was stirred for 2 h at −80 °C, then allowed to warm to −30 °C. The reaction was quenched with saturated aqueous NH_4Cl (50 mL). The phases were separated, and the aqueous layer was extracted with diethyl ether (3 × 50 mL). The combined organic extracts were dried over $MgSO_4$, filtered, and concentrated under reduced pressure. The crude product consisted of disilapropofol (**3**, 97%), 2-bromo-6-(dimethylsilyl)phenol (**9**, 1%), and 2-(dimethylsilyl)phenol (2%) as determined by ^1H NMR spectroscopy. Kugelrohr distillation (60 °C, 2.4×10^{-3} mbar) afforded disilapropofol (**3**) as a colorless liquid (1.996 g, 9.49 mmol, 82%).

^1H NMR (400.1 MHz, C_6D_6): δ = 0.26 (d, 12H, $^3J_{HH}$ = 3.7 Hz, C*H*$_3$), 4.62 (sept, 2H, $^3J_{HH}$ = 3.7 Hz, Si*H*(CH$_3$)$_2$), 5.25 (s, 1H, O*H*), 6.90 (t, 1H, $^3J_{HH}$ = 7.2 Hz, *p-H*), 7.37 (d, 2H, $^3J_{HH}$ = 7.2 Hz, *m-H*). **$^{13}C\{^1H\}$ NMR** (100.6 MHz, C_6D_6): δ = –3.8 (*C*H$_3$), 121.2 (*o-C*$_{Ar}$), 121.8 (*p-C*$_{Ar}$), 137.7 (*m-C*$_{Ar}$), 165.5 (*i-C*$_{Ar}$). **$^{29}Si\{^1H\}$ NMR** (79.5 MHz, C_6D_6): δ = –22.63 (s). **HRMS** (GC-MS): calcd. m/z for $C_{10}H_{18}OSi_2$ [M+]: 210.08907; found: 210.08932. **EA**: calcd. for $C_{10}H_{18}OSi_2$: C 57.08, H 8.62; found: C 56.62, H 8.39.

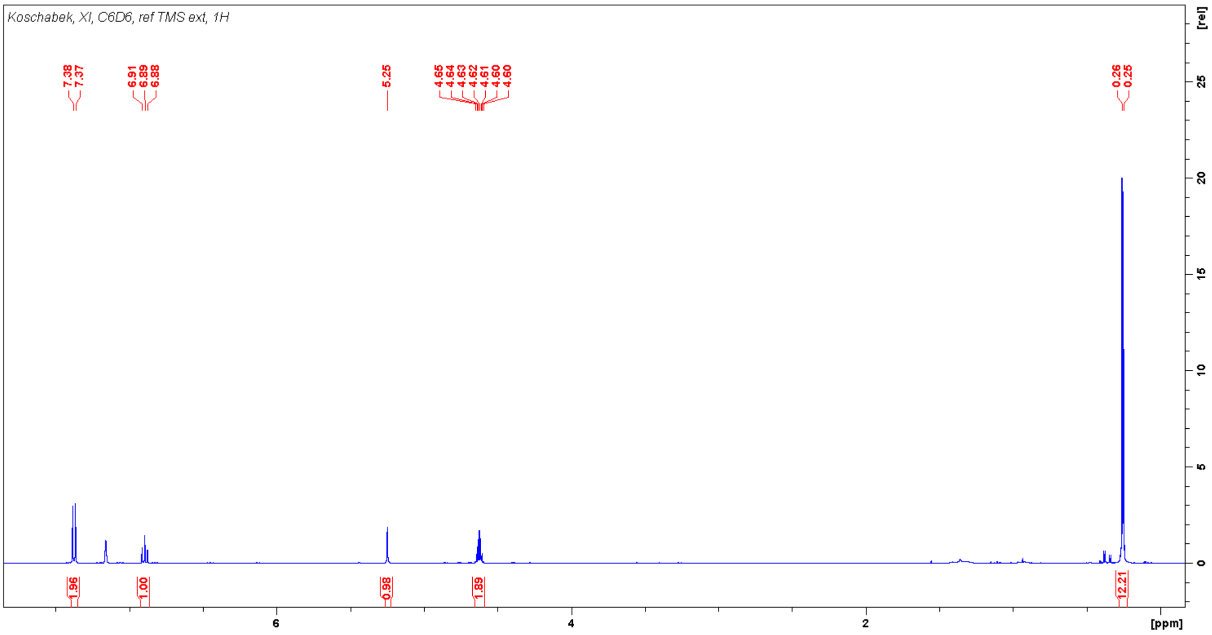

Figure S20. ^{1}H NMR spectrum (C_6D_6) of compound **3**.

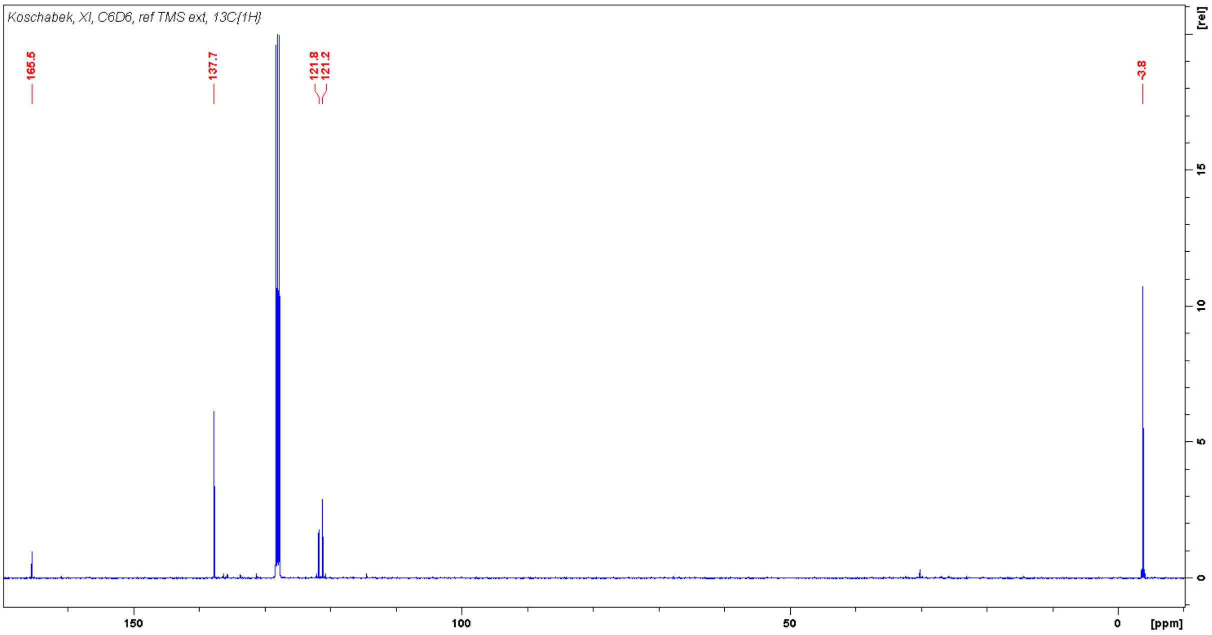

Figure S21. ^{13}C{^{1}H} NMR spectrum (C_6D_6) of compound **3**.

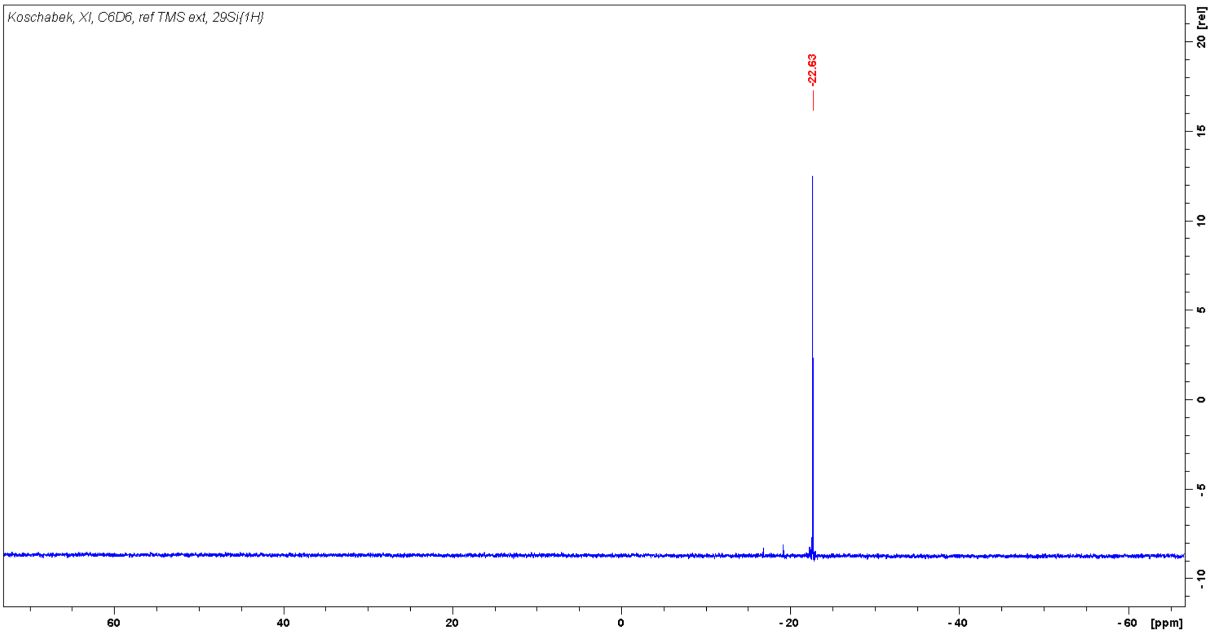

Figure S22. $^{29}Si\{^1H\}$ NMR spectrum (C_6D_6) of compound **3**.

2.10. Stability of disilapropofol (3) in aqueous solution

The stability of disilapropofol (**3**) was investigated in aqueous media by NMR spectroscopy. **3** was dissolved in 0.9% NaCl in D_2O and monitored by ^1H NMR spectroscopy at room temperature, showing no detectable decomposition over 6 weeks (Figure S23). Under mildly basic conditions (pH ≈ 8, 10 mM $NaHCO_3$ in D_2O), **3** decomposed more rapidly, with its proportion decreasing to 80% after 4 days and 30% after 2 weeks (Figure S24). Based on the NMR spectra, an exact assignment of the decomposition products cannot be made.

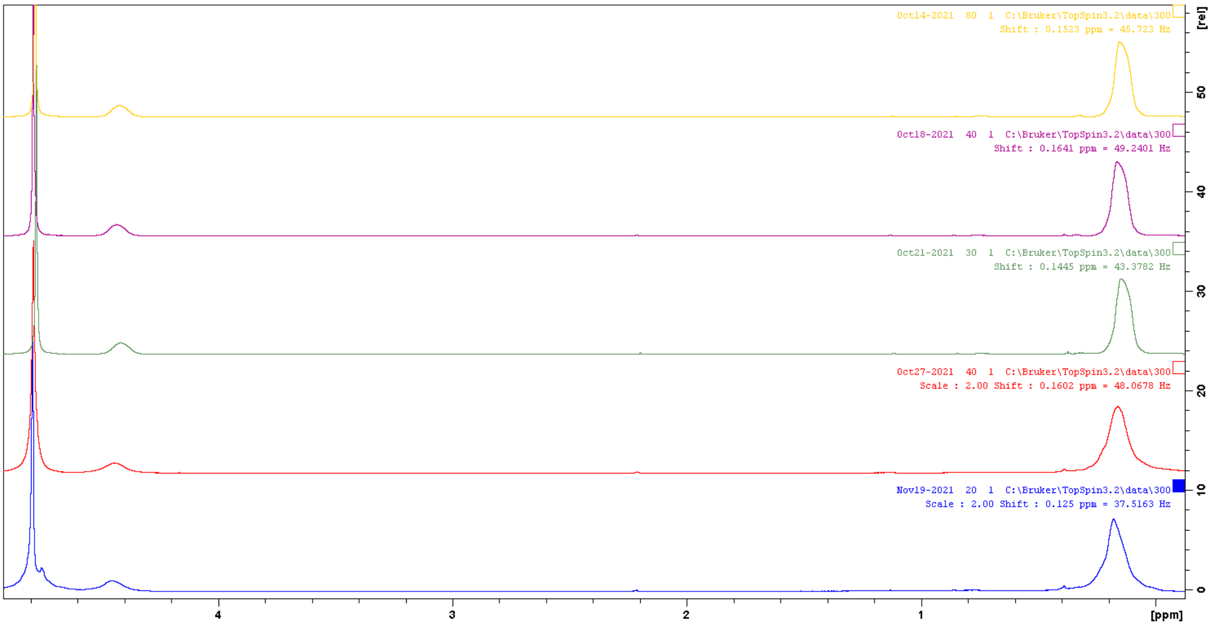

Figure S23. Stability of disilapropofol (**3**) monitored by ^1H NMR spectroscopy in D_2O containing 0.9% NaCl at room temperature. Spectra recorded on the same day (yellow), after 4 days (purple), 7 days (green), 13 days (red), and 44 days (blue).

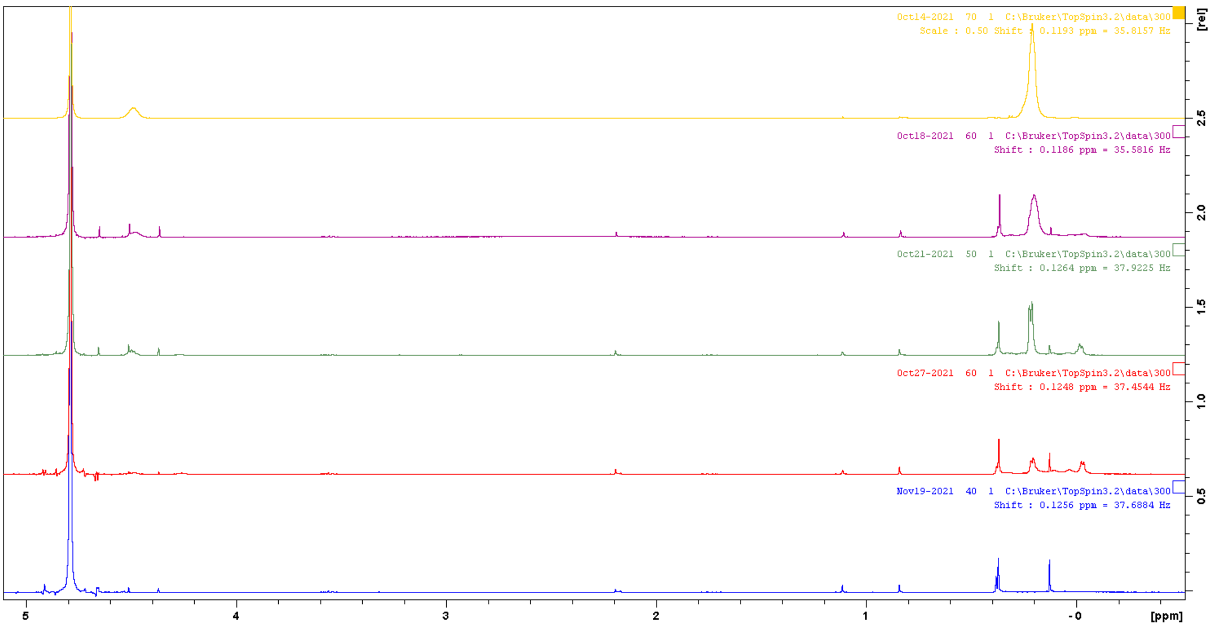

Figure S24. Stability of disilapropofol (**3**) monitored by ^{1}H NMR spectroscopy in D_2O containing 10 mM $NaHCO_3$ (pH ≈ 8) at room temperature. Spectra recorded on the same day (yellow), after 4 days (purple), 7 days (green), 13 days (red), and 44 days (blue).

3. Crystallographic analysis

Single-crystal X-ray diffraction analysis of **2-K** was performed on an XtaLAB Synergy-DW diffractometer equipped with a HyPix-Arc 150 detector at 123.00(10) K using Cu-Kα radiation (λ = 1.54184 Å). Data collection and reduction were performed using the CrysAlisPro software system, Version 1.171.41.93a.[46] The crystal structure was solved with SHELXT[47] using Olex2 (Version 1.5-alpha).[48,49] The initial structure solution of **2-K** revealed all essential molecular features. During subsequent refinements with olex2.refine[48,49] and after geometric placement of hydrogen atoms, the residual density map showed a clear presence of positive residual density around the central *iso*-propyl carbon atom and a pronounced negative residual density around the silicon atom. Therefore, the entire anion of **2-K** was split and refined as a whole molecule disorder model. After initial refinement cycles, the occupation of PART 2 was determined to be around 5%. After geometric addition of hydrogen atoms to PART 2, the potassium cation was also split to account for the residual density of the potassium atom in the structure. The same occupation constraint was chosen for PART 2 of potassium under the rationale that the flipped monosilapropofol anion most likely leads to a slight relocation of the potassium cation. Since the signal of these atoms is relatively weak and only low-resolution data up to d = 0.8 Å, no further disorder modelling was attempted. The potassium ions are surrounded by three anions of monosilapropofol in the crystal structure: two are oriented so that the negatively charged oxygen atom points toward the potassium cation (bottom of Figure S25). In contrast, the third anion coordinates side-on via the aromatic ring (see uppermost anion in Figure S25). Final refinement cycles were performed using NoSpherA2[36] employing ORCA 5.0[34,35,37] for the calculation of the electron density on a level of theory of PBE[32]/def2-TZVPP[33]. Hydrogen atom distances of PART 1 were refined using AFIX 138/AFIX 14 constraints for the carbon-bound atoms, while the silicon-bound atom was refined entirely freely. Hydrogen atoms of PART 2 were kept at the literature-reported averaged neutron distances[50] for all carbon-bound hydrogen atoms, and the silicon-bound hydrogen atom was restrained using the same distance restraint between the minor and major components. A final plot of the fractal dimensional analysis of the residual density in the unit cell according to Meindl and Henn[51] is shown in Figure S26, as calculated with Olex2 after the refinement.

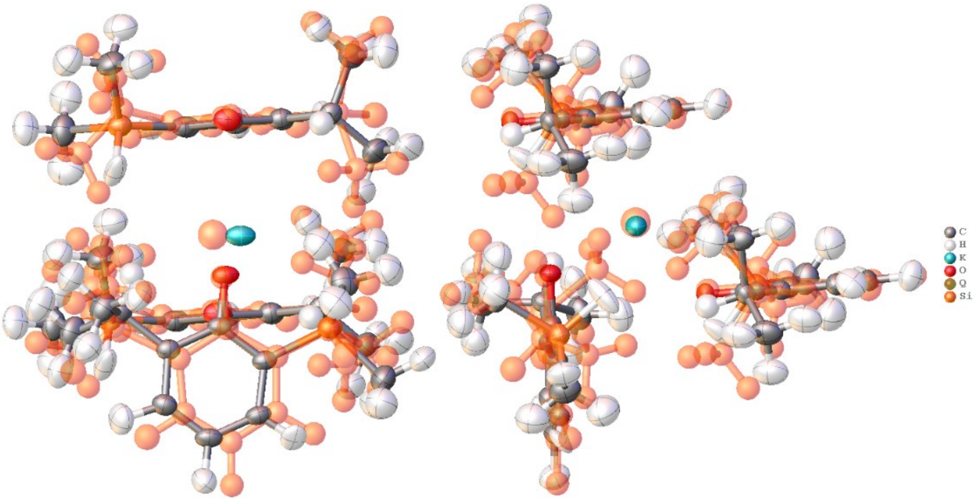

Figure S25. Visualization of the arrangement of 2-(dimethylsilyl)-6-*iso*-propylphenolate anions around the central potassium ion. Side view (left) and top view (right). The minor disorder component (PART 2) is shown in orange.

Table S3. Crystallographic details of the X-ray diffraction analysis of compound **2-K**.

Structure	Potassium 2-(dimethylsilyl)-6-*iso*-propylphenolate (**2-K**)
Space group	$P2_1$
a /Å	9.8050(2)
b /Å	6.5783(1)
c /Å	10.3691(2)
β /°	103.609(2)
V /Å^3	650.03(2)
T /K	123
Resolution /Å	0.80
Wavelength /Å	Cu-K_α
R_{int}	0.0332
Avg. redundancy	5.20
Completeness	1.00
Average I/σ	44.6
# of refln. measured	13766
# of unique refln.	2651
Criterion for observed	$F_o^2 > 2\sigma(F_o^2)$
# of observed refln.	2513
# of Params / Restr. / Constr.	301/138/19
Weighting scheme	$w = 1/(\sigma(F_o)+0.0819P^2+0.1620P)$, where $P = (F_o^2-2F_c^2)$
GooF	1.062
Final R_1	0.0430
Final wR_2	0.1164
Hooft/Flack Parameter	0.242(4)/0.258(14)
Max residual density /eÅ^{-3}	0.317
Min residual density /eÅ^{-3}	-0.440
RMS residual density /eÅ^{-3}	0.052
Disorder Occupations	PART 1: 0.949; PART 2: 0.051

NoSpherA2[36] Settings [ORCA 5.0][34,35]	PBE[32]/def-TZVPP[33], Normal Integration, CPCM(water), StrongSCF, NormalConv
CCDC deposition number	2492470

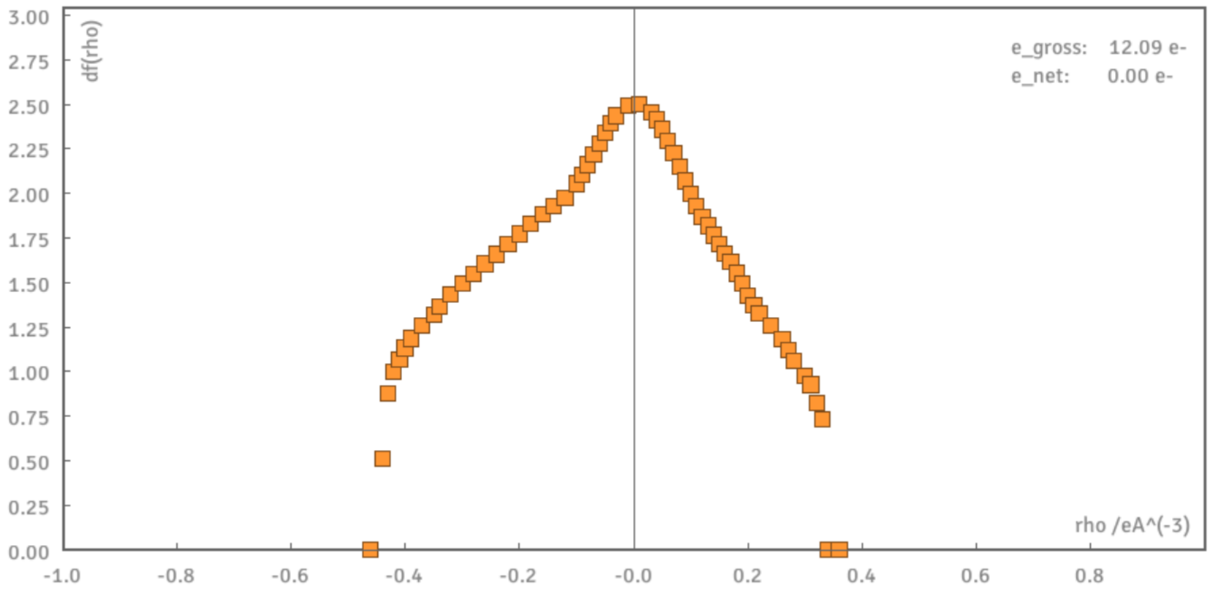

Figure S26. Fractal dimension analysis of compound **2-K** according to Meindl and Henn.[51]

4. Quantum chemical calculations

Chemical bonding analysis was performed using the atomic coordinates of the major component obtained from the NoSpherA2 crystallographic model (see Table S4). A single-point wavefunction calculation was carried out at the B3LYP/def2-TZVPP level of theory for the system with a total charge of +1. Subsequent bonding analyses were conducted using NBO 7.0[52] with the "NRT" and "BNDIDX" keywords, AIMAll[53] with default settings, and DGrid 5.0[54] with a grid spacing of 0.01 a.u. to calculate the alpha-alpha spin ELI-D and electron density for intersection analysis, yielding the Raub–Jansen index.[42] Only results relevant to the carbon–silicon-switched region are presented; additional data are available upon request.

Table S4. Atomic coordinates employed for chemical bonding analysis.

Atom type	X	Y	Z
**K**	9.381261	1.216580	8.555089
**K**	4.536959	7.432172	10.48953
**SI**	10.57354	7.801366	14.58838
**O**	9.053833	5.922526	9.558150
**C**	10.76669	6.992817	4.521266
**H**	9.039039	6.055724	5.010773
**C**	11.79793	8.868410	11.49995
**C**	11.79602	8.296194	6.882187
**C**	13.75167	10.07411	6.737515
**H**	14.41153	10.32103	4.710998
**C**	10.81334	7.632702	9.316003

**C**	13.73442	10.65024	11.23251
**H**	14.45327	11.42768	12.80194
**C**	14.73327	11.25583	8.878786
**H**	16.25418	12.55288	8.631232
**C**	12.60483	4.914731	3.626050
**H**	14.32568	6.050068	3.016899
**H**	13.19555	3.554693	5.144276
**H**	11.67443	4.112612	2.220819
**C**	12.16287	9.593272	17.17583
**H**	11.76938	12.03059	16.92331
**H**	11.58153	8.950534	19.02971
**H**	14.20014	9.521312	16.99219
**C**	10.14553	8.786744	2.377852
**H**	8.575471	10.05295	3.046507
**H**	11.98654	9.989817	1.629294
**H**	9.174726	7.685296	0.850136
**C**	11.12874	4.345798	15.13897
**H**	10.79150	4.121550	17.18473
**H**	9.515510	3.058913	13.71934
**H**	13.22721	3.871365	14.44096
**H**	7.694584	8.171622	14.86530

5. References

[46] Rigaku Oxford Diffraction, *CrysAlisPro Software System*, 2023.

[47] Sheldrick, G. M. *SHELXT* – Integrated space-group and crystal-structure determination. *Acta Crystallogr., Sect. A: Found. Adv.* **2015**, *71*, 3–8.

[48] Dolomanov, O. V.; Bourhis, L. J.; Gildea, R. J.; Howard, J. A. K.; Puschmann, H. *OLEX2*: a complete structure solution, refinement and analysis program. *J. Appl. Crystallogr.* **2009**, *42*, 339–341.

[49] Bourhis, L. J.; Dolomanov, O. V.; Gildea, R. J.; Howard, J. A. K.; Puschmann, H. The anatomy of a comprehensive constrained, restrained refinement program for the modern computing environment – *Olex2* dissected. *Acta Crystallogr., Sect. A* **2015**, *71*, 59–75.

[50] Allen, F. H.; Bruno, I. J. Bond lengths in organic and metal-organic compounds revisited: *X*—H bond lengths from neutron diffraction data. *Acta Crystallogr., Sect. B: Struct. Sci.* **2010**, *66*, 380–386.

[51] Meindl, K.; Henn, J. Foundations of residual-density analysis. *Acta Crystallogr., Sect. A: Found. Adv.* **2008**, *64*, 404–418.

[52] Glendening, E. D.; Badenhoop, J. K.; Reed, A. E.; Carpenter, J. E.; Bohmann, J. A.; Morales, C. M.; Karafiloglou, P.; Landis, C. R.; Weinhold, F. *NBO 7.0*; Theoretical Chemistry Institute and Department of Chemistry, University of Wisconsin: Madison, WI, USA, 2018.

[53] Keith, T. A. *AIMAll, Version 19.10.12*; TK Gristmill Software: Overland Park, KS, USA, 2019.

[54] Kohout, M. *DGrid, Version 5.0*; Max Planck Institute for Chemical Physics of Solids: Dresden, Germany, 2019.
